# Tailoring polymer acceptors by electron linkers for achieving efficient and stable all-polymer solar cells

**DOI:** 10.1093/nsr/nwab151

**Published:** 2021-08-16

**Authors:** Qiang Wu, Wei Wang, Yao Wu, Rui Sun, Jing Guo, Mumin Shi, Jie Min

**Affiliations:** The Institute for Advanced Studies, Wuhan University, Wuhan 430072, China; The Institute for Advanced Studies, Wuhan University, Wuhan 430072, China; The Institute for Advanced Studies, Wuhan University, Wuhan 430072, China; The Institute for Advanced Studies, Wuhan University, Wuhan 430072, China; The Institute for Advanced Studies, Wuhan University, Wuhan 430072, China; The Institute for Advanced Studies, Wuhan University, Wuhan 430072, China; The Institute for Advanced Studies, Wuhan University, Wuhan 430072, China; Key Laboratory of Materials Processing and Mold (Zhengzhou University), Ministry of Education, Zhengzhou 450002, China; Institute of Polymer Optoelectronic Materials and Devices, State Key Laboratory of Luminescent Materials and Devices, South China University of Technology, Guangzhou 510640, China

**Keywords:** all-polymer solar cells, polymer acceptors, electron linker, intermolecular interaction, molecular compatibility, stability

## Abstract

The trade-off between efficiency and stability is a bit vague, and it can be tricky to precisely control the bulk morphology to simultaneously improve device efficiency and stability. Herein, three fused-ring conducted polymer acceptors containing furan, thiophene and selenophene as the electron linkers in their conjugated backbones, namely PY-O, PY-S and PY-Se, were designed and synthesized. The electron linker engineering affects the intermolecular interactions of relative polymer acceptors and their charge transport properties. Furthermore, excellent material compatibility was achieved when PY-Se was blended with polymer donor PBDB-T, resulting in nanoscale domains with favorable phase separation. The optimized PBDB-T : PY-Se blend not only exhibits maximum performance with a power conversion efficiency of 15.48%, which is much higher than those of PBDB-T : PY-O (9.80%) and PBDB-T : PY-S (14.16%) devices, but also shows better storage and operational stabilities, and mechanical robustness. This work demonstrates that precise modification of electron linkers can be a practical way to simultaneously actualize molecular crystallinity and phase miscibility for improving the performance of all-polymer solar cells, showing practical significance.

## Teaser

This paper demonstrates that electron linker engineering can be a practical strategy to fine-tune intermolecular interactions and molecular miscibility for simultaneously improving the efficiency and stability of all-polymer solar cells.

## INTRODUCTION

Solution-processed bulk-heterojunction (BHJ) polymer solar cells (PSCs) composed of *p*-type conjugated polymer donors (*P*_D_s) blended with *n*-type small molecule non-fullerene acceptors (SM-NFAs) or conjugated polymer acceptors (*P*_A_s) have made significant efficiency improvements, with power conversion efficiencies (PCEs) rapidly improving from 14% to over 17% for NFA-based PSCs [1–6] and from 11% to over 15% for all-polymer solar cells (all-PSCs) [7–11] over the past two years, respectively. Extensive research into control of specific aspects of the materials such as electronic energy levels, optical bandgaps and intermolecular interactions, has resulted in the availability of a myriad of photovoltaic materials for PSC applications [2,12–14]. In contrast, stability studies have been deprioritized because they often produce unsatisfactory results. This is because the resulting finely mixed, and phase-separated regions in the delicate BHJ structure are typically metastable [15,16], which is generally far away from the thermodynamic equilibrium, resulting in rapid performance attenuation of devices for many intrinsic factors (e.g. molecular structure [17–19], donor/acceptor (D/A) compatibility [20] and molecule migration [21], etc.) and external stresses (e.g. irradiation [22,23], heating [15,24] and mechanical stress [25,26], etc.). For instance, many organic compounds chemically degrade under light and oxygen conditions [27], and the blend morphology of active layers can evolve via molecular dimerization and migration [28], crystallization and/or phase segregation under extended exposure to heat, illumination and thermal cycling conditions [15]. Thus, it is important to emphasize that continued progress in SM-NFA- and all-polymer-based PSCs is still challenging, even though their efficiencies are approaching the required threshold considered for commercial viability [29,30].

Several specific approaches such as designing organic photovoltaic materials (e.g. suitably extending the conjugated planarity of molecules [31] and reducing the crystallinity of photovoltaic polymers [17]), modifying the degree of polymerization [19], selecting suitable D/A pairs [32], cross-linking between D/A components [33] and incorporating solid additives into the active layer [15] have been demonstrated to partially solve specific stability issues of BHJ active layers, especially in terms of storage stability [33], photostability [17,19] and thermal stability [31]. Among these strategies, designing active layer materials is perhaps the most impactful way to balance potential trade-offs between achieving desirable photovoltaic properties and introducing instability in BHJ micromorphology. In other words, the molecular geometry and intermolecular packing of photovoltaic materials are important considerations to keep high-performance PSCs under environmental operation conditions.

There is a common consensus that compared to SM-NFA-based PSCs, all-polymer systems are considered to present more potential for practical applications because of their low molecular diffusion coefficients, remarkable operational stability and thermal stability, and robust mechanical properties [11,34–39]. For instance, some research groups have demonstrated that all-polymer systems based on poly{[N,N^′^-bis(2-octyldodecyl)-naphthalene1,4,5,8-bis(dicarboximide)-2,6-diyl]-alt-5,5^′^-(2,2^′^-bithiophene)} (N2200) or its derivatives as *P*_A_s exhibited better storage stability and thermal stability compared to the corresponding SM-NFAs-based blend morphologies [39–41]. In addition, the PM6 : PY-S (PY-S) all-polymer system, reported in our previous works [7,26], also showed better storage stability and mechanical stability than those of the PM6 : Y5-C20-based system. Despite this, it is worth noting that the PY-S-based all-PSCs also showed a remarkable reduction in PCE in a short period of hundreds of hours, with just ∼76% of their initial efficiencies retained. Some highly efficient all-polymer systems reported by us with PCEs >14% also showed unfavorable environmental stabilities [36,42]. Undeniably, bulky and low-planar *P*_A_ materials compared to SM-NFAs are more desirable from a stability standpoint. However, the stability metrics for all-polymer systems, especially in terms of the BHJ morphological stability, will be determined on a case-by-case basis as these are dependent on the micromorphology of D/A materials originating from relevant intermolecular interactions of *P*_D_s or *P*_A_s. This is because, on the one hand, intermolecular interactions need to be sufficiently strong to form suitably phase-separated interpenetrating networks and to facilitate exciton dissociation and charge transport. On the other hand, intermolecular interactions should not be so strong as to invite molecular crystallite aggregates, molecular rearrangement and increased vertical disorder in active layers during operation. As such, there is an urgent need for further endeavors towards the intermolecular interactions of designed photovoltaic materials and to find promising approaches to effectively fine-tune the strength of intermolecular forces and also to find matching materials to carefully modify molecular compatibility of donor and acceptor materials to meet the stability requirements of high-performance PSC applications.

A plausible avenue is to tune the electron linker in conjugated backbones, which can effectively fine-tune intermolecular interactions. Based on this assumption, herein we demonstrate such a methodology using a highly efficient Y5-C20-derivative *P*_A_s and changing the electron linkers (furan, thiophene and selenophene) to form a new series of fused-ring conducted *P*_A_s, namely PY-O, PY-S [7,26] and PY-Se. The three synthesized *P*_A_s exhibit comparable optical and electrochemical properties, but PY-Se possesses stronger crystallinity behavior in the solid-state compared to the PY-O and PY-S *P*_A_s. Furthermore, a PY-Se-based active layer is demonstrated to benefit both crystallinity in blends and miscibility with a medium band-gap *P*_D_ PBDB-T (Poly[(2,6-(4,8-bis(5-(2-ethylhexyl)thiophen-2-yl)-benzo[1,2-b:4,5-b^′^]dithiophene))-alt-(5,5-(1^′^,3^′^-di-2-thienyl-5^′^,7^′^-bis(2-ethylhexyl)benzo[1^′^,2^′^-c:4^′^,5^′^-c^′^]dithiophene-4,8-dione)]). As a result, PBDB-T : PY-Se devices possessed a much higher PCE of 15.48% compared to the PY-O- (9.80%) and PY-S-based devices (14.16%). The PBDB-T : PY-Se blend also showed better storage and operational stabilities and higher tensile strength. This study illustrates that modification of electron linkers could be a promising strategy to alter π–π stacking interaction of the polymer acceptors and fine-tune their molecular miscibility with a specific *P*_D_.

## RESULTS AND DISCUSSION

**Figure 1.**
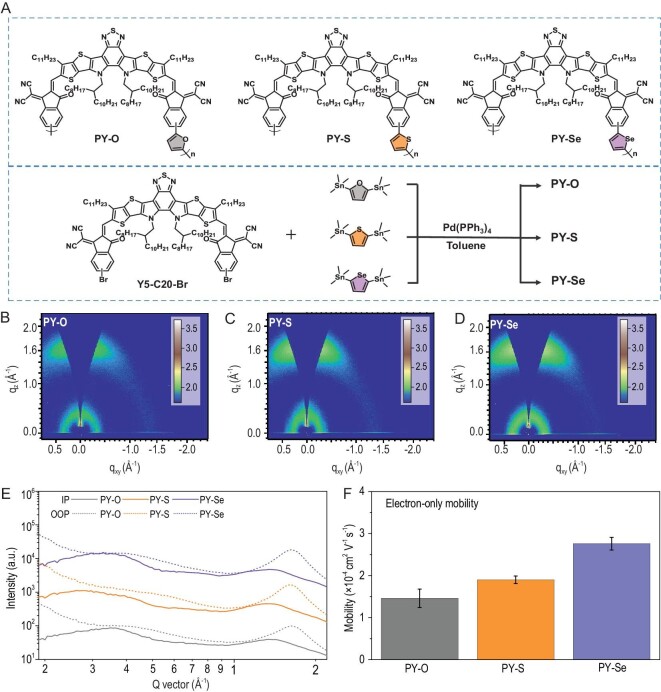
(A) Molecular structures of PY-O, PY-S and PY-Se as well as their synthetic routes. 2D GIWAXS profiles for the (B) PY-O, (C) PY-S, and (D) PY-Se pristine films. (E) The 1D GIWAXS line curves with respect to the OOP and IP directions, acquired at a critical incident angle of 0.13°. (F) The electron mobilities of the pristine *P*_A_ films.

To effectively fine-tune the intermolecular interactions, a series of *P*_A_s (PY-O, PY-S and PY-Se; Fig. 1A) were designed by polymerization of Y5-C20 building blocks and incorporation of different electron linkers (furan (O), thiophene (S) and selenophene (Se)). Compound Y5-C20-Br was synthesized via a published method [26]. The synthesis routes of the three *P*_A_s are outlined in Fig. 1A. Characterization information and detailed synthesis are also provided in the Supplementary data (Experimental section). The *M*_w_ distribution plots of these three *P*_A_s are shown in Figs S1–S3. Weight-average molecular weights (*M*_w_s) of obtained *P*_A_s are between 18.2 and 20.7 kg mol^–1^, and polydispersity indexes (PDIs) are between 1.9 and 2.0 (Table S1). These data were determined by high-performance gel chromatography with polystyrene standards. Notably, these comparable *M*_w_s and PDIs allow direct comparison of the material properties independent of the degree of *P*_A_ polymerization [39].

The normalized absorption spectra of these three *P*_A_s in chloroform solution and solid film are exhibited in Fig. S4, and cyclic voltammograms are presented in Fig. S5. The corresponding optical and electrochemical parameters are summarized in Table S1. In the solutions, the absorption peak (*λ*_max_^sol^) increases from 756 nm of PY-O to 776 nm of PY-S, and further increases to 783 nm of PY-Se. The corresponding *P*_A_s also show linearly increasing maximum film absorption wavelength (*λ*_max_^film^) and onset wavelength (*λ*_edge_) values according to the sequential chalcogen elements. Those indicate that heterocycles containing S and Se as electron linkers, as compared to the furan aromatic linker, can enhance the backbone interactions in the *P*_A_s. Of note is that the electron push-pull properties of the molecular backbones of these three *P*_A_s can also affect their absorption spectra in solutions. In addition, the most significant absorption feature of PY-Se is the maximum absorption coefficient of 1.03 × 10^5^ cm^−1^ in the solid state, which is higher than those of PY-O (0.95 × 10^5^ cm^−1^) and PY-S (1.01 × 10^5^ cm^−1^) neat films. Besides, all of the *P*_A_s with different chalcogen heterocycles exhibit well-matched lowest unoccupied molecular orbital/highest occupied molecular orbital (LUMO/HOMO) energy levels with PBDB-T, with enough offsets for effective charge transfer, as depicted in Fig. S5B. Notably, the comparable LUMO/HOMO values of the designed *P*_A_s suggest that the corresponding chalcogen heterocycles as electron linkers have little influence in energy levels. In addition, molecular simulation was carried out using density functional theory (DFT) with B3LYP/6–31G (d, p) basis set. In particular, the HOMO and LUMO levels and related electron distributions were calculated (Figs S6–S8). The trend of variation for molecular orbital energy levels is consistent with the results obtained from the CV tests (Table S1).

To shed light on the effects of the electron linkers on the molecular-packing arrangements in the solid-state, we conducted two-dimensional grazing-incidence wide-angle X-ray scattering (2D-GIWAXS) measurements. Figure 1B–D presents 2D-GIWAXS patterns of the *P*_A_ films (PY-O, PY-S and PY-Se), and the relevant crystallographic parameters of these pristine films are presented in Table S2. All of the *P*_A_s adopt a preferential face-on orientation exhibiting similar prominent (010) diffraction peaks located at *q*_z_ = 1.63 Å^−1^ in the out-of-plane (OOP) direction. As with the above-discussed optical properties, the crystalline correlation lengths (CCLs) of these three *P*_A_s also exhibit a linear increase (CCL_PY-O_ = 16.71 Å, CCL_PY-S_ = 18.05 Å, CCL_PY-Se_ = 18.42 Å) according to the sequential chalcogen elements. This result reflects the crystallinity behaviors of the neat *P*_A_ films and the strength of intermolecular interactions in the solid-state and also implies their charge transport properties [43,44]. Thus, the electron mobilities of the neat *P*_A_s films were further measured using the space-charge-limited-current (SCLC) method (Fig. S9). The electron mobility of PY-Se is 2.76 $ \times $10^–4^ cm^2^ V^–1^ s^–1^, slightly higher than those of the pristine PY-O (1.48$ \times $10^–4^ cm^2^ V^–1^ s^–1^) and PY-S (1.90 $ \times $ 10^–4^ cm^2^ V^–1^ s^–1^) films, as depicted in Fig. 1F. These results suggest that PY-Se with a selenophene as an electron linker shows enhanced aggregation strength and better molecular crystallinity in the thin film compared to the PY-O and PY-S *P*_A_s, indicating that the electron linker engineering can effectively modify the intermolecular interactions of *P*_A_s.

**Figure 2.**
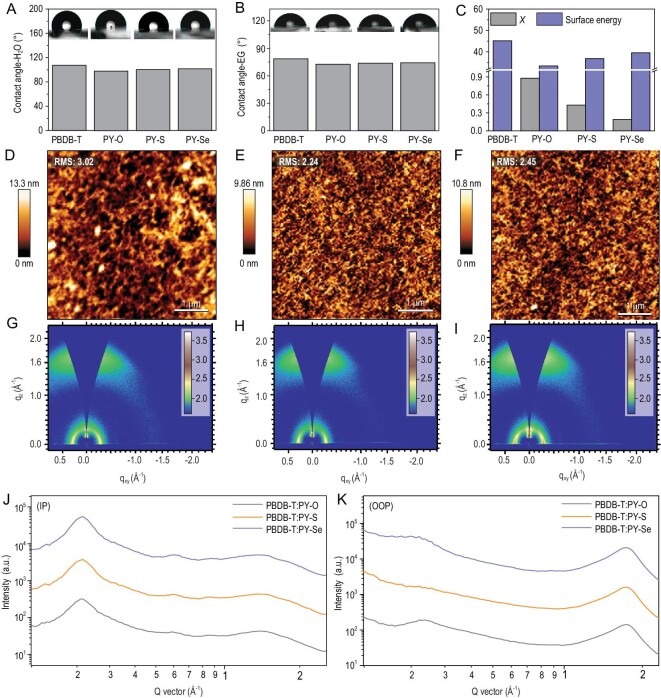
Water (A) and ethylene glycol (B) contact angles measured on surfaces of the neat films. (C) Surface energy of the neat films and Flory-Huggins interaction parameter (χ) values of relevant all-polymer systems based on PBDB-T as donor. AFM phase images of (D) PBDB-T : PY-O, (E) PBDB-T : PY-S and (F) PBDB-T : PY-Se blend films. GIWAXS patterns for blend films of (G) PBDB-T : PY-O, (H) PBDB-T : PY-S and (I) PBDB-T : PY-Se. All images were corrected for monitor and film thickness and displayed on the same logarithmic color scale. (J) IP and (K) OOP profiles acquired at a critical incident angle of 0.13°.

To further investigate the molecular compatibility of donor and acceptor materials, we used water (Fig. 2A) and ethylene glycol (EG, Fig. 2B) to conduct surface energy measurements of these three *P*_A_s and PBDB-T introduced as *P*_D_ in this work (see Fig. S10). As shown in Fig. 2C, the corresponding surface energy values are 33.37 mN m^–1^ for PY-O, 36.80 mN m^–1^ for PY-S, 39.52 mN m^–1^ for PY-S and 45.14 mN m^–1^ for PBDB-T. Thus, the Flory-Huggins interaction parameter (χ) values between donor and acceptors, are calculated from experimentally measured contact angles (Fig. S10), and summarized in Table S3. As a direct consequence (see Fig. 2C), the calculated χ values for PY-O : PBDB-T, PY-S : PBDB-T and PY-Se : PBDB-T were 0.88, 0.43 and 0.19, respectively. A high χ value of 0.88 implies severe phase aggregation in the PBDB-T : PY-O system, which was illustrated by atomic force microscope (AFM) measurements. As shown in Fig. 2D, the PBDB-T : PY-O film shows formation of separated domains with a bi-continuous interpenetrating network. Note that the large domain sizes on the length scale of hundreds of nanometers in the PBDB-T : PY-O blend cause poor charge separation process, resulting from the longer distance for exciton diffusions, which will be discussed further below. In contrast, AFM images of the PBDB-T : PY-S (Fig. 2E) and PBDB-T : PY-Se (Fig. 2F) films exhibit significantly better developed bi-continuous interpenetrating networks with nm-scale domains, explaining their improved *J*_SC_ and FF values.

To further investigate the crystallization at nanoscale, 2D-GIWAXS measurements of pristine PBDB-T film (Fig. S11) and its blend films with different *P*_A_s (Fig. 2G–I) were carried out. The relevant crystallographic parameters of these films are summarized in Table S4. For the pristine PBDB-T film, the polymer backbone preferred an obvious face-on orientation relative to the substrate, supported by the prominent (010) reflection peak at 1.69 Å^−1^ (crystal coherence length, CCL = 28.56 Å) in the OOP direction and lamellar (100) peak at 0.294 Å^−1^ (d_100_ = 21.37) in the IP direction. After blending with PBDB-T, all the blends show obvious face-on orientation relative to the substrate. In the IP direction (Fig. 2J), these all-polymer blend films exhibit obvious (100) diffraction peaks at a similar position of ∼0.29 Å^–1^. The PBDB-T : PY-S and PBDB-T : PY-Se blend films show a slightly smaller lamellar stacking distance of 21.30 Å than that of 21.67 Å for the PBDB-T : PY-O blend. Additionally, the PBDB-T : PY-S and PBDB-T : PY-Se blend films show much higher CCL values (17.40 Å and 17.75 Å) than that of 16.98 Å for the PBDB-T : PY-O blend in the OOP direction (Fig. 2K), while the PBDB-T : PY-Se blend film has the highest CCL of 17.75 Å among these three blends. Although the AFM images demonstrate analysis of the χ values in these three systems, the PBDB-T : PY-O film possesses inferior CCL in the OOP direction, indicating its large phase separation with low phase purity and high D : A mixture region. Overall, the PBDB-T : PY-Se blend shows better phase separation and more favorable blend microstructure with improved molecular ordering, leading to enhanced photovoltaic performance as compared to the PY-O- and PY-S-based all-PSCs.

**Figure 3.**
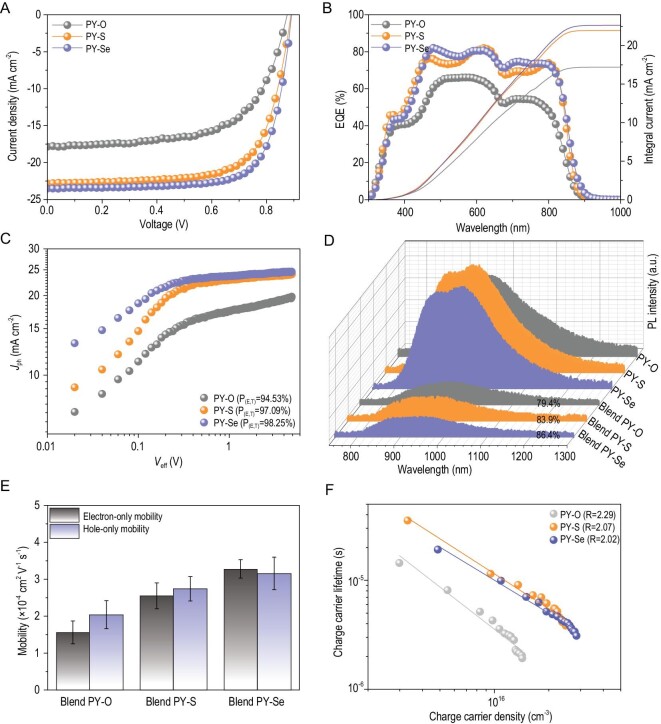
(A) *J–V* characteristics of the best PSCs under the illumination of AM 1.5 G, 100 mW cm^−2^. (B) The corresponding EQE spectra of devices. (C) Characteristics of the photocurrent density versus effective voltage (*J*_ph_–*V*_eff_). (D) PL spectra of the pristine acceptors (PY-X (O, S, Se)) and corresponding blend films. The intensities are corrected by their absorptions at the excitation wavelength (639 nm). (E) The electron and hole mobilities of the devices based on the corresponding blends. (F) Charge carrier lifetime *τ*, obtained from TPV, as a function of charge density *n*, calculated from CE under *V*_OC_ conditions (from 0.15 to 2.50 suns).

The effect of electron linkers on photovoltaic properties was comprehensively studied from the designed all-PSCs with a conventional architecture of indium tin oxide (ITO)/poly(3,4-ethylenedioxythiophene) : poly(styrenesulfonate) (PEDOT : PSS)/PBDB-T : PY-X (O, S, Se)/Poly[(9,9-bis(3^′^-(N,N-dimethyl)-nethylammoinium-propyl)-2,7-fluorene)-alt-2,7-(9,9-dioctylfluorene)]dibromide (PFN-Br)/100 nm Ag. All the all-PSCs underwent the same process optimization because of their structural similarities, in which active layers with a thickness of ∼100 nm were obtained from a spin-coated blend solution of chloroform : 1-chloronaphthalene (CN) with a D/A ratio (w/w) of 1 : 1.2 and a total solid concentration of 16 mg mL^–1^. Detailed processing parameters of these all-polymer systems are described in the Supplementary data. The optimization details of these all-polymer systems are provided in Figs S12–S15, and the photovoltaic parameters are summarized in Tables S5–S8. Figure 3A provides the current density-voltage (*J − V*) curves of the corresponding best-performing all-PSCs based on different *P*_A_s. Impressively, the PBDB-T : PY-Se device yields a PCE as high as 15.48%, with an open-circuit voltage (*V*_OC_) of 0.891 V, a short-circuit current density (*J*_SC_) of 23.52 mA cm^–2^, along with a fill factor (FF) of 73.85%. Note that this device efficiency (15.48%) is higher than that (13.80%) of the previously reported all-PSCs based on the PY-Se derivative (PFY-1Se) as acceptor and PBDB-T as donor [45]. In addition, compared to the PY-Se-based all-PSCs, the PY-O- and PY-S-based devices exhibit lower PCEs of 9.80% (with *V*_OC_ of 0.876 V, *J*_SC_ of 17.86 mA cm^–2^, FF of 62.68%) and 14.16% (with *V*_OC_ of 0.889 V, *J*_SC_ of 22.84 mA cm^–2^, FF of 69.71%), respectively, as presented in Table 1. The higher PCEs for the PBDB-T : PY-Se system are attributed to improvements in all photovoltaic parameters. Although these three systems show comparable energy levels, their *V*_OC_ values are slightly different. The above-mentioned blend morphologies of these systems may lead to associated energetic loss mechanisms (Fig. S16) and thus cause slight *V*_OC_ variation (Table 1). External quantum efficiency (EQE) measurements of these systems, as plotted in Fig. 3B, were carried out to explain the difference in the measured *J*_SC_ values from *J − V* plots.

**Table 1.** Optimized photovoltaic performances of the all-PSCs based on PBDB-T/acceptors, measured under one sun illumination.

	*V*_OC_	*J*_SC_	*J*_SC, EQE_ ^a^	FF	PCE (PCE^b^)
PBDB-T:acceptor	(V)	(mA cm^–2^)	(mA cm^–2^)	(%)	(%)
PY-O	0.876	17.86	17.20	62.68	9.80 (± 0.34)
PY-S	0.889	22.84	21.98	69.71	14.16 (± 0.26)
PY-Se	0.891	23.52	22.65	73.85	15.48 (± 0.31)

^a^ *J*_SC_, _EQE_ represents the integrated current density obtained from EQE spectra; ^b^the average PCE values with standard deviations were obtained from 12 devices.

To clarify the larger difference of *J*_SC_ and FF values, we studied the charge photogeneration of the three all-PSCs. The photocurrent density (*J*_ph_) versus the internal voltage (*V*_in_) curves of the devices are shown in Fig. 3C. This result indicates that the high *J*_SC_ and FF values obtained for PBDB-T : PY-S and PBDB-T : PY-Se systems result from the charge collection being efficient enough at the internal electric field. In contrast, the PBDB-T : PY-O device did not exhibit an apparent saturation regime for *J*_ph_ even at high *V*_in_ (> 1 V), which is mainly attributed to a decrease in limited charge extraction and recombination [46]. We further investigated the *J*_ph_ at high *V*_in_ regimes (*V*_in_ = 4 V), 19.34 mA cm^–2^, 23.85 mA cm^–2^ and 24.54 mA cm^–2^ for the PY-X (O, S, Se)-based all-polymer devices, respectively. As just a small portion of the large *J*_SC_ and *J*_ph_ losses of PBDB-T : PY-O device compared to the PBDB-T : PY-S and PBDB-T : PY-Se systems can be partially explained by the weaker absorption coefficient of the PBDB-T : PY-O blend (Fig. S16), an inferior *J*_ph_ of 19.34 mA cm^–2^ for the PBDB-T : PY-O device indicates that its charge extraction (CE) is much poorer, as shown by the transient photocurrent (TPC) curves of these devices measured under light intensity closing to one sun illumination. As exhibited in Fig. S17, the extraction time was calculated to be *τ* = 0.96 μs for the PBDB-T : PY-O device, *τ* = 0.50 μs for the PBDB-T : PY-S device and *τ* = 0.43 μs for the PBDB-T : PY-Se device, respectively. The increase of extracted charge carriers at longer timescales indicates the unfavorable states or domains that can act as traps for charge carriers in the PBDB-T : PY-O devices, resulting in poor CE property.

Based on this point, photoluminescence (PL) spectra were further used to study the effects of the electron linkers in the *P*_A_s on exciton dissociation and charge transport properties in these blends. As shown in Fig. 3D, the PL emission of acceptors is quenched 79.4% in the PY-O-based blend, 83.9% in the PY-S-based blend and 86.4% in the PY-Se-based blend, respectively. This result illustrates that the exciton dissociation of the PBDB-T : PY-O blend is a vital limiting factor for the lower *J*_SC_ as compared to the other two systems. Additionally, the hole and electron mobilities of these three systems were investigated by analyzing the *J − V* characteristics of single-carrier devices (Fig. S18 for hole-only mobilities and Fig. S19 for electron-only mobilities, respectively). As depicted in Fig. 3E, the PBDB-T : PY-Se blends show more-balanced hole- and electron-mobilities of 3.16 $ \times $ 10^–4^ cm^2^ V^–1^ s^–1^ and 3.28 $ \times $ 10^–4^ cm^2^ V^–1^ s^–1^ in devices compared to the PBDB-T : PY-S system (a *μ*_h_ of 2.74$ \times $10^–4^ cm^2^ V^–1^ s^–1^ and a *μ*_e_ of 2.55 $ \times $ 10^–4^ cm^2^ V^–1^ s^–1^) and PBDB-T : PY-O system (a *μ*_h_ of 2.04 $ \times $ 10^–4^ cm^2^ V^–1^ s^–1^ and a *μ*_e_ of 1.56 $ \times $ 10^–4^ cm^2^ V^–1^ s^–1^). Notably, the low and unbalanced electron and hole mobilities of the optimized PBDB-T : PY-O system indicate that its blend is transport-limited, also supported by the above-mentioned *J*_ph_ analysis (Fig. 3C).

It is worth noting that the high and balanced charge transport properties in devices generally lead to reduced carrier recombination losses. Using the transient photovoltage (TPV) and CE techniques, which can depict the charge carrier lifetime *τ* (Fig. S20) as a function of charge carrier density *n* (Fig. S21) under open-circuit conditions, *τ*(n), we shed light on the differences of carrier recombination mechanisms in these all-polymer systems. As exhibited in Fig. 3F, a lower recombination order value *R* (*R* = 2.02), which was calculated via the equation of *τ* = *τ*_0_(n_0_/n)^λ^ (where *τ*_0_ and n_0_ are constants and λ is the so-called recombination exponent) [47], was found for the PBDB-T : PY-Se device as compared to PBDB-T : PY-O (*R* = 2.29) and PBDB-T : PY-S (*R* = 2.07). The non-radiative voltage losses of these systems calculated from the electroluminescence EQE (Fig. S22) are 0.335 eV for the PBDB-T : PY-O, 0.327 eV for the PBDB-T : PY-S and 0.323 eV for the PBDB-T : PY-Se, respectively. This result indicates that there is a reduced non-radiative energy loss that contributes to the improved *V*_OC_ in the PBDB-T : PY- Se devices. Thus, all the results as discussed above indicate improved photovoltaic parameters in the PBDB-T : PY-Se devices.

**Figure 4.**
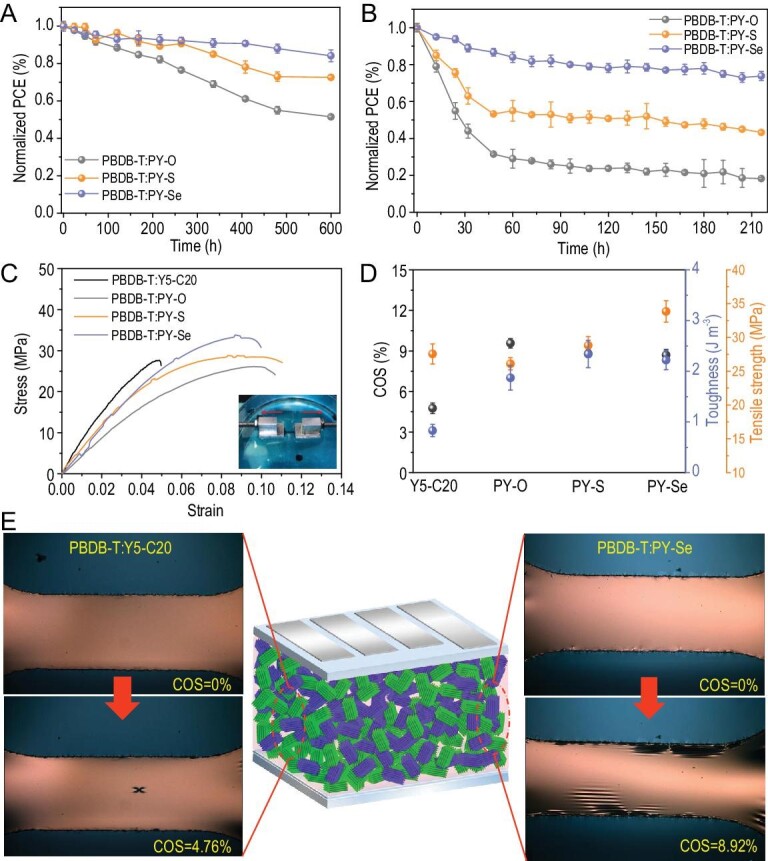
Storage stability and operational stability of all-polymer solar cell devices based on different *P*_A_s kept at room temperature (A) and under one sun illumination (B) in N_2_-filled glovebox. The values are summarized from four cells. The mechanical robustness properties of the active layers under a pseudo free-standing tensile test system and the related statistical values from five specimens. (C) Representative stress-strain curves. (D) Elastic modulus, elongation, toughness and tensile modules of PY-X (O, S, Se)-based blend films and PBDB-T : Y5-C20 blend film. (E) Optical microscopy images of PBDB-T : Y5-C20 film and PBDB-T : PY-Se optimized films conducted under different strains.

The relationships between molecular structure and morphological stability were emphatically studied to perfect the potential assessment of the investigated *P*_A_s based on different electron linkers. We firstly explored the long-time stored stability of the corresponding devices tested in a nitrogen glove box at room temperature. As shown in Fig. 4A, the PY-O- and PY-S-based devices exhibited inferior storage stability to that of the PY-Se-based device. Their performances decreased to 51.5% and 72.5%, respectively, of their initial efficiencies after 600 h storage, while the PBDB-T : PY-Se device decreased to only 84.3% PCE loss within the same time frame. This degradation trend of stored devices is identical to the attenuation trend of the devices exposed to light stress, as presented in Fig. 4B. After continuous light-soaking, PY-O- and PY-S-based films showed significant light-induced losses within 216 hours (75.29% and 81.75%), while PY-Se-based film was 85.03% of quenching efficiency over the same period. Light-induced degradation affects all the photovoltaic parameters (see Fig. S23). This trend is further confirmed by the change in PL intensity (Figs S24 and S25). The initial PL quenching rates of these blends were decreased after light-soaking for 216 hours, with PY-O- and PY-S-based blends gaining PL intensity more accelerated than the PY-Se-based system, underlining that PY-Se is thermally more stable.

We further demonstrated that the stability of photoactive layers could be quickly and reliably analyzed by measuring the space-charge-limited current of hole-only or electron-only devices under illumination (Figs S26 and S27). This indicates that the performance degradation probably originates from decreased and increasingly unbalanced electron and hole mobilities (Table S9). To further gain insight into the charge recombination behaviors after light-soaking, charge carrier lifetime (Fig. S28) as a function of charge carrier density (Fig. S29) for the all-polymer solar cells was investigated (Fig. S30). For the PBDB-T : PY-Se devices, the recombination order (R) slightly increased from 2.02 for the fresh devices (0 h) to 2.08 for the corresponding device under one sun illumination for 216 h. In contrast, the *R* value of PBDB-T : PY-O devices significantly increased from 2.29 for the fresh devices (0 h) to 2.53 for the corresponding device under one sun illumination for 216 h (Table S9). Increased recombination order in OSCs can be linked to trap-mediated recombination and/or reduced mobility. All these physical characterizations as mentioned above suggest that the PBDB-T : PY-Se system shows much more stable blend microstructure.

Additionally, to develop a quantitative understanding of the mechanical stabilities or properties of all-polymer systems depending on acceptor types, we further employed a pseudo-free-standing tensile test on a water surface that can directly yield stress-strain (*S-S*) curves of mechanical properties (Fig. 4C). The detailed mechanical values, including elastic modulus, crack-onset strain (COS), toughness and tensile modules for the blend films are summarized in Table S10. Compared to the small molecule Y5-C20-based blend with a COS of 4.76%$ \pm $0.39%, all the all-polymer systems show higher elongation values of 8.70%$ \pm $0.42%–9.57%$ \pm $0.35%. The excellent mechanical stability of the all-polymer blends was also confirmed by calculation of toughness. As presented in Fig. 4D, a remarkable contrast in the toughness values of the all-polymer blends (1.87$ \pm $0.24–2.34$ \pm $0.27 J m^–3^) and the Y5-C20-based blend (0.83$ \pm $0.12 J m^–3^) was found, resulting from increased acceptor chain length by polymerization. The tensile behaviors of the Y5-C20- and PY-Se-based blends were compared using optical microscopy during the tensile tests, as depicted in Fig. 4E, indicating that there is a dramatic difference in their fracture response under tensile strain. Notably, as compared to the PY-S- and PY-Se-based blends, PY-O-based film shows a relatively low fracture toughness. It is mainly attributed to the low miscibility of PBDB-T and PY-O (Fig. 2C), resulting in limited chain entanglement and large domain sizes in the blend (Fig. 2D) [48]. In contrast, the improved mechanical properties of the PY-S- and PY-Se blends can be attributed primarily to the ductility of the polymer films imposed by the entangled polymer chains and their bi-continuous interpenetrating networks with nm-scale domains. Of particular note is the increased stress at the same strain in the PY-Se-based blend compared with PY-S, as a result of improved hardness of the crystalline domains resulting from its stronger intermolecular interactions.

**Figure 5.**
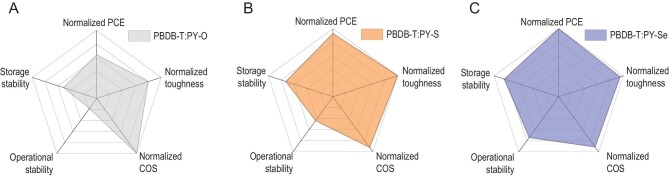
Radar chart visualization of the multivariate data analysis of (A) PBDB-T : PY-O, (B) PBDB-T : PY-S and (C) PBDB-T : PY-Se. Data have five variables : storage stability, operational stability, COS, toughness and PCE. The shadow area represents the comprehensive performance of a determined system. The range of coordinates from the center to outside of each axis in the radar chart is 0–100%. Note that the COS and toughness values are normalized by the highest values among these three systems.

Previously, it was found that electron linkers can regulate intermolecular arrangement and crystallinity, thus affecting blend morphology and device efficiency [49,50]. The relationship between intermolecular interactions and phase separation in blends is generally considered in most cases [34,49–51], but the effects of D/A compatibility as well as their intermolecular interactions on relevant stability issues are often neglected. In this work, we systematically elucidated the detailed influence of the electron linkers on efficiency and stability of the investigated all-polymer systems and summarized the corresponding results using visualized radar charts (Fig. 5)—a straightforward approach but the start of thinking about how we provide a comprehensive evaluation of design strategy.

As provided in Fig. 5, we can conclude that the higher degree of molecular crystallinity found for PY-Se because of its strong intermolecular interactions is reflected in enhanced stabilities compared to the other two systems. This finding for the morphological degradation rate of the active layer as a function of intermolecular interaction energy is consistent with all our experimental results as mentioned above. It already allows for quite deep insight into the fundamental mechanisms behind electron linker engineering. In addition, *P*_D_ − *P*_A_ compatibility (or phase miscibility) not only determines the blend morphological characteristics (Fig. 3), thus affecting the device efficiency and stability [52,53] (Fig. 2A; Fig. 4A and B), but also influences the mechanical robustness of relevant active layers, strongly supported by tensile test results (Fig. 4D). It seems plausible that the molecular crystallinity and phase compatibility are not directly related, supported by our analysis results, which is inconsistent with some previous findings [35,54,55], especially in N2200-based systems [56–58]. Nonetheless, we can conclude with care that both intermolecular interactions and D-A compatibility simultaneously determined the blend morphological characteristics, which result in the performance differences of all-polymer systems based on various electron linkers (Fig. 5).

## CONCLUSION

In summary, a series of narrow band-gap polymer acceptors PY-X (O, S, Se) containing furan, thiophene and selenophene as the electron linkers in their conjugated backbones were designed and synthesized for application in all-PSCs. The electron linker engineering significantly affects the physical and chemical properties and intermolecular interactions of relative *P*_A_s and their charge transport properties. A PBDB-T : PY-Se system with remarkable D/A compatibility showed maximum performance with a PCE of 15.48%, much higher than those of PBDB-T : PY-O (9.80%) and PBDB-T : PY-S (14.16%) devices, supported by the optimized bulk microstructure with respect to its physical mechanisms in parallel. Note that the achieved PCE value (15.48%) is also one of the highest values in the all-PSCs reported. Additionally, the PY-Se-based blend displayed much higher storage stability and light-soaking stability than those of the other two systems. Better toughness values have also been realized in the PBDB-T : PY-Se blend, mainly resulting from suitable D/A compatibility for achieving favorable domains with nanoscale phase separation and meanwhile maintaining relatively stable morphology with suitable intermolecular interactions. Of particular note is the in-depth analysis of the effect of electron linkers on intermolecular interactions and molecular miscibility and its influence on BHJ morphology and device performance. The strategy of precise modification of electron linkers could be a practical way to simultaneously actualize molecular crystallinity and phase miscibility for improving the performance of all-polymer solar cells, showing practical significance.

## Supplementary Material

nwab151_Supplemental_File
