# Supplementary material for: Tailoring polymer acceptors by electron linkers for achieving efficient and stable all-polymer solar cells
Source: Natl Sci Rev. 2021 Aug 16;9(2):nwab151. doi: 10.1093/nsr/nwab151 (PMC8824755; doi:10.1093/nsr/nwab151)
Supplement: nwab151_Supplemental_File [file nwab151_supplemental_file.docx]

Supporting Information

1. Experimental Section

**1.1 Materials**

- 1. *Materials*. PBDB-T, and PFN-Br were purchased from Solarmer Materials Inc. The aqueous dispersion of PEDOT:PSS was purchased from Heraeus, Germany. Chloroform was dried and distilled from appropriate drying agents prior to use. The synthetic route of the conjugated polymer acceptor PY-S (PYT) was synthesized in our lab, as reported previously.^1^
  2. **Supplementary Scheme 1. Synthetic procedures of polymer acceptors PY-O、PY-S and PY-Se.**

In a dry 25 mL two-necked flask, Y5-C20-Br (187.4 mg, 0.10 mmol) and 2,5-bis(trimethylstannyl)furan (39.3 mg, 0.10 mmol) were dissolved in 10 mL dry toluene. After flushing with argon for 20 minutes, 7.6 mg of Pd(PPh3)4 was added into the flask quickly, the mixture was flushed with argon for another 20 minutes and stirred at 110 ℃ for 150 minutes, respectively. Then the reaction mixture was cooled down to room temperature and precipitated into 200 mL of methanol. The polymer was collected by filtration through a Soxhlet extractor and then subjected to Soxhlet extractions with methanol, hexane, acetone, and chloroform each for 12 hours, respectively. Then the chloroform fraction was concentrated and precipitated with methanol, the purple-black solid was obtained PY-O (114 mg, 64% yield).

In a dry 25 mL two-necked flask, Y5-C20-Br (187.4 mg, 0.10 mmol) and 2,5-bis(trimethylstannyl)thiophene (41.0 mg, 0.10 mmol) were dissolved in 10 mL dry toluene. After flushing with argon for 20 minutes, 7.6 mg of Pd(PPh3)4 was added into the flask quickly, the mixture was flushed with argon for another 20 minutes and stirred at 110 ℃ for 150 minutes, respectively. Then the reaction mixture was cooled down to room temperature and precipitated into 200 mL of methanol. The polymer was collected by filtration through a Soxhlet extractor and then subjected to Soxhlet extractions with methanol, hexane, acetone, and chloroform each for 12 hours, respectively. Then the chloroform fraction was concentrated and precipitated with methanol, the purple-black solid was obtained PY-S (121 mg, 67% yield).

In a dry 25 mL two-necked flask, Y5-C20-Br (187.4 mg, 0.10 mmol) and 2,5-bis(trimethylstannyl)selenophene (45.7 mg, 0.10 mmol) were dissolved in 10 mL dry toluene. After flushing with argon for 20 minutes, 7.6 mg of Pd(PPh3)4 was added into the flask quickly, the mixture was flushed with argon for another 20 minutes and stirred at 110 ℃ for 50 minutes, respectively. Then the reaction mixture was cooled down to room temperature and precipitated into 200 mL of methanol. The polymer was collected by filtration through a Soxhlet extractor and then subjected to Soxhlet extractions with methanol, hexane, acetone, and chloroform each for 12 hours, respectively. Then the chloroform fraction was concentrated and precipitated with methanol, the purple-black solid was obtained PY-Se (94.2 mg, 51% yield).

- 1. **Instruments and measurements**

^1^H NMR and ^13^C NMR spectra were recorded on a JNM-ECZ400 400 MHz NMR spectrometer at room temperature. Chemical shifts are reported in parts per million (ppm, δ). ^1^H NMR was referenced to tetramethylsilane (0 ppm) or solvent residual peak (CDCl_3_: ^1^H NMR: 7.26 ppm). Thermogravimetric analysis (TGA) was carried out using TG 209F1 (NETZSCH) instrument with a heating rate of 10 ℃ min^-1^ under nitrogen atmosphere.

Electrochemical properties were studied by cyclic voltammetry (CV). CV was performed on a CS350H electrochemical workstation with a conventional three-electrode system in a tetrabutylammonium hexafluorophosphate (*n*-Bu_4_NPF_6_, 0.1 M) acetonitrile solution at a scan rate of 100 mV s^-1^. Glassy carbon disc coated with sample film was used as the working electrode. A Pt wire was used as the counter electrode, and Ag/AgCl was used as the reference electrode. The HOMO and LUMO energy levels (*E*_HOMO_/*E*_LUMO_) can be calculated from the onset oxidation/reduction potentials (*ϕ*_ox_/*ϕ*_red_) in the cyclic voltammograms according to the equations of *E*_HOMO_/*E*_LUMO_ = -e (*ϕ*_ox_/*ϕ*_red_ + 4.8 - *ϕ*_Fc/Fc_^+^) (eV), where *ϕ*_Fc/Fc_^+^ is the redox potential of ferrocene/ferrocenium (Fc/Fc+) couple in the electrochemical measurement system, and the energy level of Fc/Fc^+^ was taken as -4.8 eV below the vacuum level.

**1.4 Device Fabrication and Testing**

Solar cells were fabricated in the configuration of the traditional sandwhich structure with an indium tin oxide (ITO, South China Science & Technology Co. Ltd.) glass positive electrode and a poly[(9,9-bis(3′-(N,N-dimethyl)-nethylammoinium-propyl)-2,7-fluorene)-alt-2,7(9,9dioctylfluorene)]dibromide (PFN-Br) / silver (Ag) negative electrode. The ITO-based substrates were pre-cleaned in an ultrasonic bath of detergent, deionized water, acetone, and isopropanol, and UV-treated in the ultraviolet-ozone chamber (Jelight Company, USA) for 15 min. A thin layer of PEDOT:PSS (poly(3,4-ethylene dioxythiophene): poly(styrene sulfonate)) was filtered through a 0.45 μm poly(tetrafluoroethylene) (PTFE) filter and spin-coated at 4000 rpm for 30s on the ITO substrate. Subsequently, PEDOT:PSS film was baked at 150 ºC for 15 min in the air, and the thickness of the PEDOT:PSS layer is approximately 40 nm. For the fabrication of bulk heterojunction (BHJ) OSCs, A mixture of PBDB-T: PYX (O, S, Se) all-polymer blend solutions was dissolved in CF/CN mix solvent (16 mg mL^−1^ in total) with stirring for at least 12 h. Then, these blend solutions were spin-coated on the PEDOT:PSS layer to form active layers. The post-processing is then further optimized, as detailed in the following sections. The optimized films were thermally annealed at 110℃ for 10 min in glovebox. The thicknesses of the active layers are about ~100 nm. The thickness of the photoactive layer is measured by a surface profiler (Alpha-Step 500, KLA-Tencor, USA). For all types of devices, a methanol solution of PFN-Br at a concentration of 0.5 mg mL^-1^ was spin-coated onto the photoactive layer at 3000 r.p.m. for 30 s. Finally, the top Ag electrode of 100 nm thickness was evaporated in vacuum onto the cathode buffer layer at a pressure of 5×10^-6^ mbar. The typical active area of the investigated devices was 5 mm^2^.

The current-voltage characteristics of the solar cells were measured under AM 1.5 G irradiation on an Enli Solar simulator (100 mW cm^-2^). Before each test, the solar simulator was calibrated with a standard single-crystal Si solar cell (made by Enli Technology Co., Ltd., Taiwan, calibrated by The National Institute of Metrology (NIM) of China). Short circuit currents under AM1.5 G (100 mW cm^-2^) conditions were estimated from the spectral response and convolution with the solar spectrum. The external quantum efficiency was measured by a Solar Cell Spectral Response Measurement System QE-R3011 (Enli Technology Co., Ltd.). A calibrated silicon detector was used to determine the absolute photosensitivity at different wavelengths.

**1.5 Space charge limited current (SCLC) measurements**

Single carrier devices were fabricated, and the dark current-voltage characteristics were measured and analyzed in the space charge limited current (SCLC) regime following the references. The structure of hole-only devices was Glass/ITO/PEDOT:PSS/active layer/MoO_3_ (15 nm)/Ag (100 nm). For the electron only devices, the structure was Glass/ITO/ZnO/active layer/PFN-Br/Ag (100 nm), where Ag was evaporated. Mobilities were extracted by fitting the current density-voltage curves using the Mott-Gurney relationship. The reported mobility data are average values over the six devices of each sample.

**1.6 Morphology measurements**

Atomic Force Microscopy (AFM) images were obtained by using Nano Wizard 4 atomic force microscopy (JPK Inc. Germany) in QI mode to observe the surface morphologies of the different films deposited on glass substrates. For the probe, we select the Triangular pyramid probe (BRUKER, NCHV-A model). The cantilever parameters are: T is 4 μm; L is 125 μm; W is 40 μm; *f_0_* is 320 Hz, and *k* is 42 N m^-1^. During the process of observation, the scan speed is 58.2 μm/s, and the size is 5$\times$5 μm^2^.

Grazing-incidence Wide-Angle X-ray Scattering (GIWAXS) measurements were conducted on a Xenocs-SAXS/WAXS system with X-ray wavelength of 1.5418 Å. The film samples were irradiated at a fixed angle of 0.13^o^.

**1.7 Physical tests**

*Photoluminescence (PL) and electroluminescence (EL) measurements*: PL data were collected using a Zolix Flex One Spectrometer. The PL excitation wavelength was set to 639 nm.

*Transient photovoltage (TPV) measurements*: For TPV measurements, devices were directly connected to an oscilloscope in open-circuit conditions (1MΩ). Then the device was illuminated with a white light LED at different light intensities. A small optical perturbation was applied using a 405 nm laser-diode which was adjusted in light intensity to produce a voltage perturbation of $\Delta Vo <10 mV \ll Voc$. The amount of charges generated by the pulse was obtained by integrating a photocurrent measurement (50 Ω) without bias light.

*Charge extraction (CE) measurements*: For CE measurements, it can be used to determine the charge density in the active layer of the device at any point in the *J*-*V* curve. The devices were held at a specified voltage in the dark or under illumination. At a certain time t_0_ the light is switched off, the cell is switched to short-circuit conditions, and the resulting current transient is recorded with an oscilloscope. Most of the charge is extracted in a few microseconds due to a high internal electrical field at short circuit conditions. In addition, a fast analog switch from Texas Instruments (TS5A23159) is used to perform the switching from the specified voltage to short circuit conditions. It provides a very quick switching time (50 ns), a low on-state resistance (1 Ω), high off-state resistance (> 1MΩ) and a very low charge injection (<< 10^15^ cm^2^V^-1^s^-1^). A Keithley 2440 source-measurement unit is used to set the initial device voltage.

*Transient photocurrent (TPC) measurements*: Relevant solar cells were excited with a 405 nm laser diode. The transient photocurrent response of the devices at short circuit conditions to a 200 𝜇s square pulse from the LED with no background illumination. The current traces were recorded on a Tektronix DPO3034 digital oscilloscope by measuring the voltage drop over a 5-ohm sensor resistor in series with the solar cell. DC voltage was applied to the solar cell with an MRF544 bipolar junction transistor in a common collector amplifier configuration.

- 1. **Stability measurements**

We performed the long-time stored experiments of the unencapsulated all-polymer devices investigated in this study, measured under dark conditions and light stability under one sun illumination at room temperature in N2-filled glovebox for 216 hours. In details, the long-term stability of unencapsulated devices was conducted with a glove-box integrated multichannel solar cell performance decay test system fabricated by our group under a testing condition in accordance with ISOS-L-1. The devices were put inside a nitrogen-filled glovebox (H_2_O < 1 ppm, O_2_ < 1 ppm) and continuously illuminated with a white LED array (XLamp CXA1512 6500K CCT). The illumination light intensity was initially set before testing to make sure the output short-circuit current density equals the value that was measured under standard conditions mentioned earlier, and it monitored by a photodiode (Hamamtsu S1336-8BQ) to guarantee stable light intensity. *J-V* characters of the devices were checked periodically, and the photovoltaic parameters were calculated automatically according to the achieved *J-V* curves. Notably, the photovoltaic parameters of devices under illumination were recorded overtime automatically and the degradation curves were shown. The cell temperature was measured occasionally, and the temperature range during aging was approximately 30 ^o^C. The solar cells were fabricated in a glovebox and aged in the same conditions, excluding the well-known effects of oxygen and water degradation from our experiments.

**1.9 Pseudo free-standing tensile test.**

For the tensile testing specimen, the active layers were spin-coated onto the PEDOT:PSS/glass substrate. The active layer specimen with a size of 1.2×0.5 cm was prepared by using a cutting plotter (JW-20W-KS, Wuhan Junwei Tech. Co., LTD). To float the specimen on the water surface, water was allowed to penetrate into the PEDOT:PSS layer. Subsequently, PEDOT: PSS was dissolved, and the active layer was delaminated from the glass substrate. By performing this process at the water surface, the floating active layer specimen could be obtained. Specimen gripping was achieved by attaching PDMS-coated Al grips on the specimen gripping areas using van der Waals adhesion. The tensile test was performed by a linear stage with a strain rate of 0.001 mm/s. During the tensile test, stress and strain data were obtained through a load cell and a digital image correlation (DIC) (ShenZhen Zolix Sensor Technology Co., Ltd.) device, respectively. All of tensile tests were carried out under the ambient conditions (Temperature ~ 25 ℃, relative humidity (RH) ~ 30 %).

**1.10 Experimental data (Figures and Tables)**

**Table S1**. The optical and electrochemical parameters of the investigated polymer acceptors as well as their degrees of polymerization.

| Polymer acceptors | Molecular weights (*M*_w_) | PDIs | Solution^a^  *λ*_max_ (nm) | Film^b^  *λ*_max_ (nm) | *E*_g_^opt c^  (eV) | HOMO^d^  (eV) | LUMO^d^  (eV) | *E*_g_^cv^  (eV) |
| --- | --- | --- | --- | --- | --- | --- | --- | --- |
| PY-O | 19.2 kDa | 2.00 | 756 | 793/855 | 1.45 | -5.57 | -3.78 | 1.79 |
| PY-S | 20.7 kDa | 1.90 | 776 | 801/864 | 1.44 | -5.55 | -3.78 | 1.77 |
| PY-Se | 18.2 kDa | 2.03 | 783 | 793/874 | 1.42 | -5.56 | -3.77 | 1.79 |

*^a^*Measured in chloroform solution. *^b^*Cast from chloroform solution. *^c^*Bandgap estimated from the onset wavelength (*λ*_edge_) of the optical absorption: *E*_g_^opt^ = 1240/*λ*_edge_. ^d^Measured by electrochemical cyclic voltammetry.

**Figure S1.** The molecular weight distribution plot of polymer acceptor PY-O.

**Figure S2.** The molecular weight distribution plot of polymer acceptor PY-S.

**Figure S3.** The molecular weight distribution plot of polymer acceptor PY-Se.


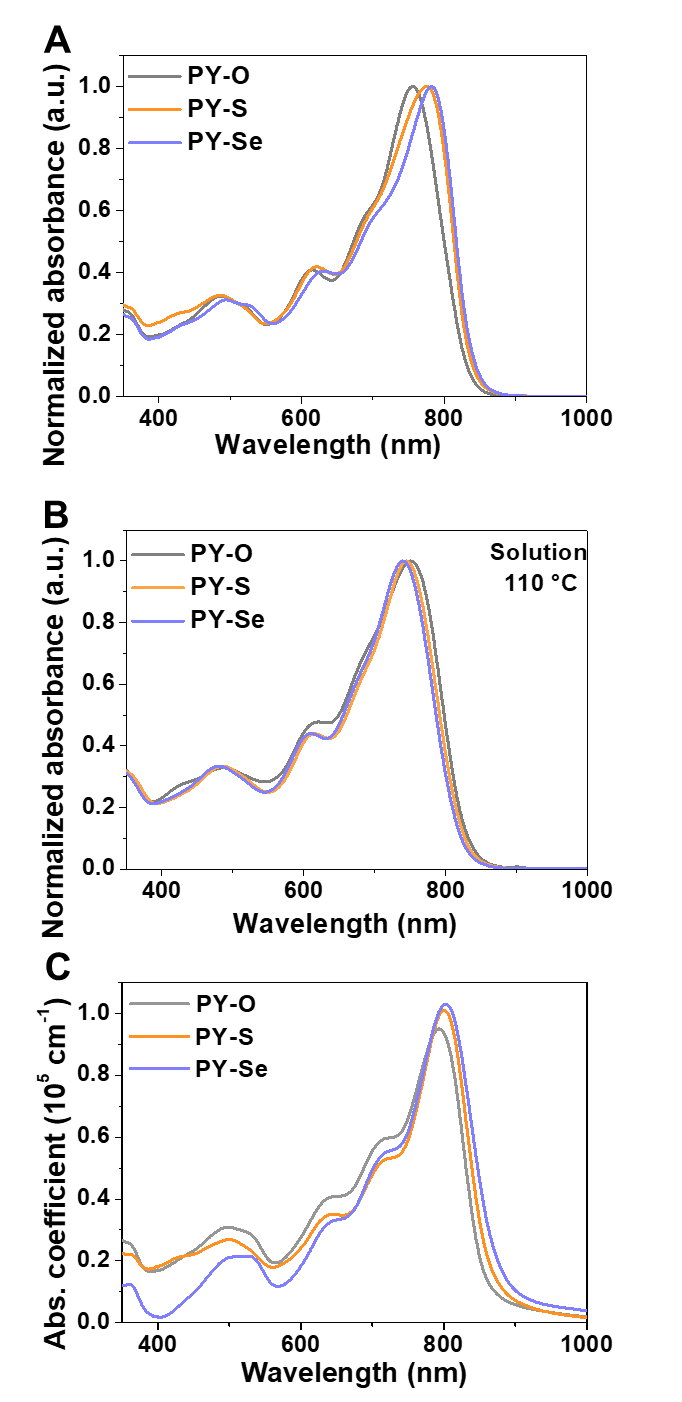


**Figure S4.** UV-Vis absorption spectra of the investigated polymer acceptors (A) in diluted chloroform solutions, (B) in diluted 110 ℃ chlorobenzene solution, and (C) in the films casted from chloroform solutions, respectively.


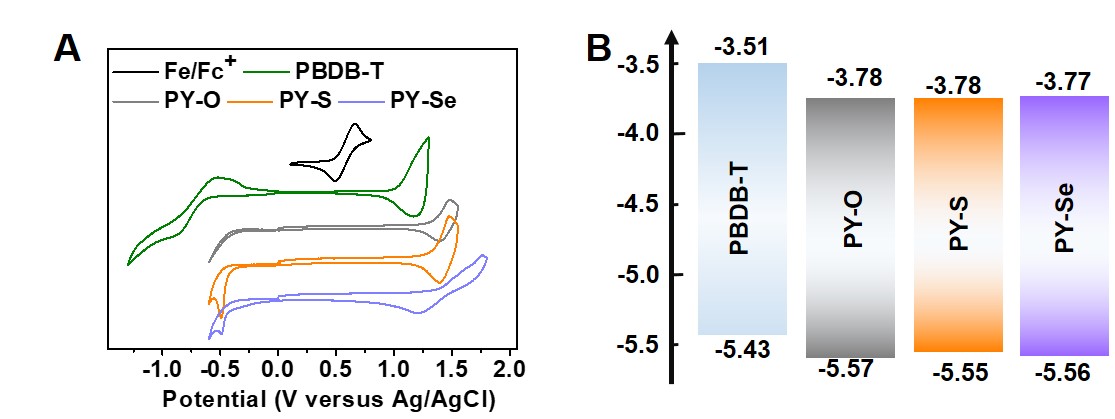


**Figure S5**. (A) Cyclic voltammogram (CV) curves of the PBDB-T and *P*_A_s (PY-O, PY-S and PY-Se) films on glassy carbon electrode in 0.1 mol L^-1^ Bu_4_NPF_6_ acetonitrile solution at a scan rate of 100 mV s^-1^. (B) The energy levels of relevant active layer materials.


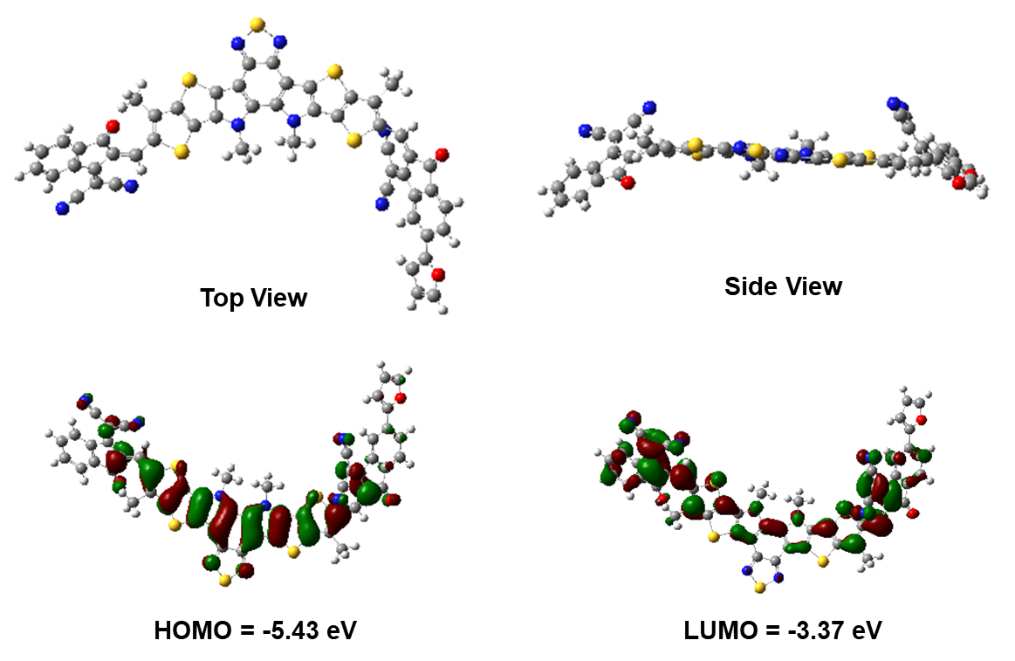


**Figure S6**. Molecular geometry, LUMO and HOMO energy levels of PY-O calculated by DFT/B3LYP/6-31G (d, p).


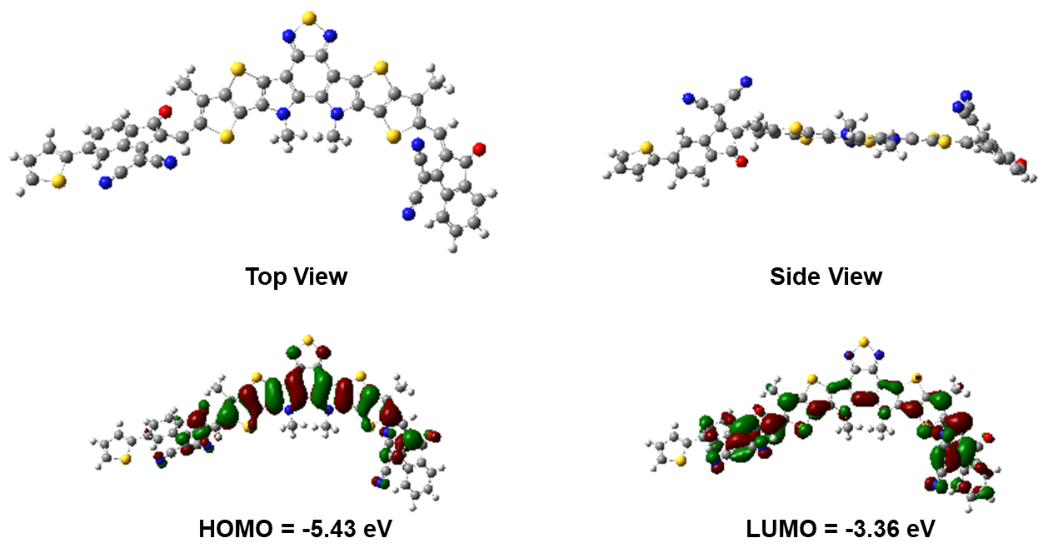


**Figure S7**. Molecular geometry, LUMO and HOMO energy levels of PY-S calculated by DFT/B3LYP/6-31G (d, p).


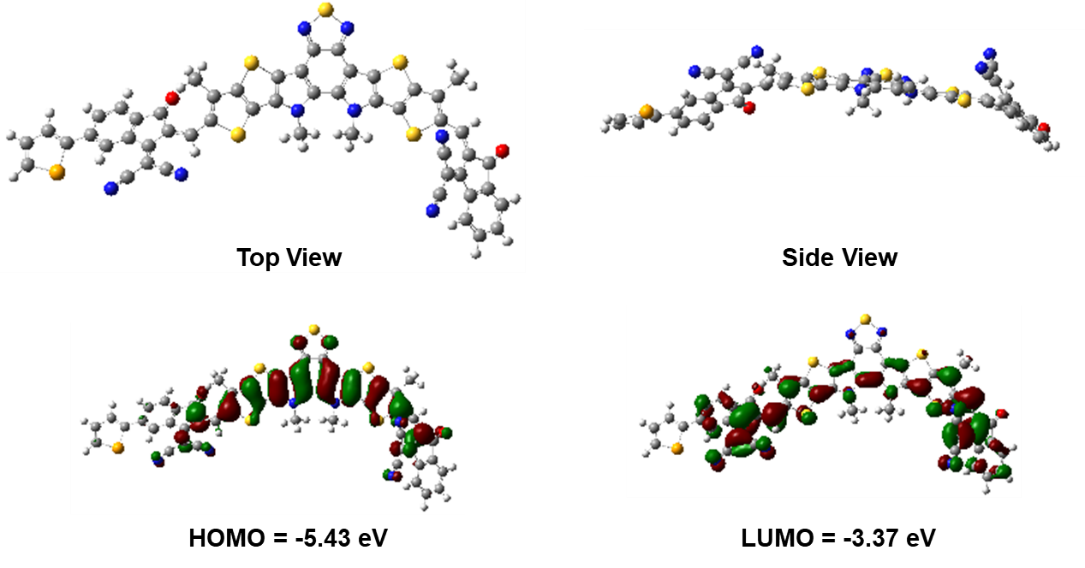


**Figure S8**. Molecular geometry, LUMO and HOMO energy levels of PY-Se calculated by DFT/B3LYP/6-31G (d, p).

**Table S2**. Summary of the extracted data from the 2D-GIWAXS patterns of the pristine *P*_A_ films, including PYF, PYT and PYS.

| Samples | IP (100) | | OOP (010) | | |
| --- | --- | --- | --- | --- | --- |
|  | Q value  (Å^-1^) | Lattice spacing  (Å) | | FWHM  (Å^-1^) | Coherence length  (Å) |
| PY-O | 0.357 | 17.60 | | 0.376 | 16.71 |
| PY-S | 0.280 | 22.44 | | 0.348 | 18.05 |
| PY-Se | 0.321 | 19.57 | | 0.341 | 18.42 |


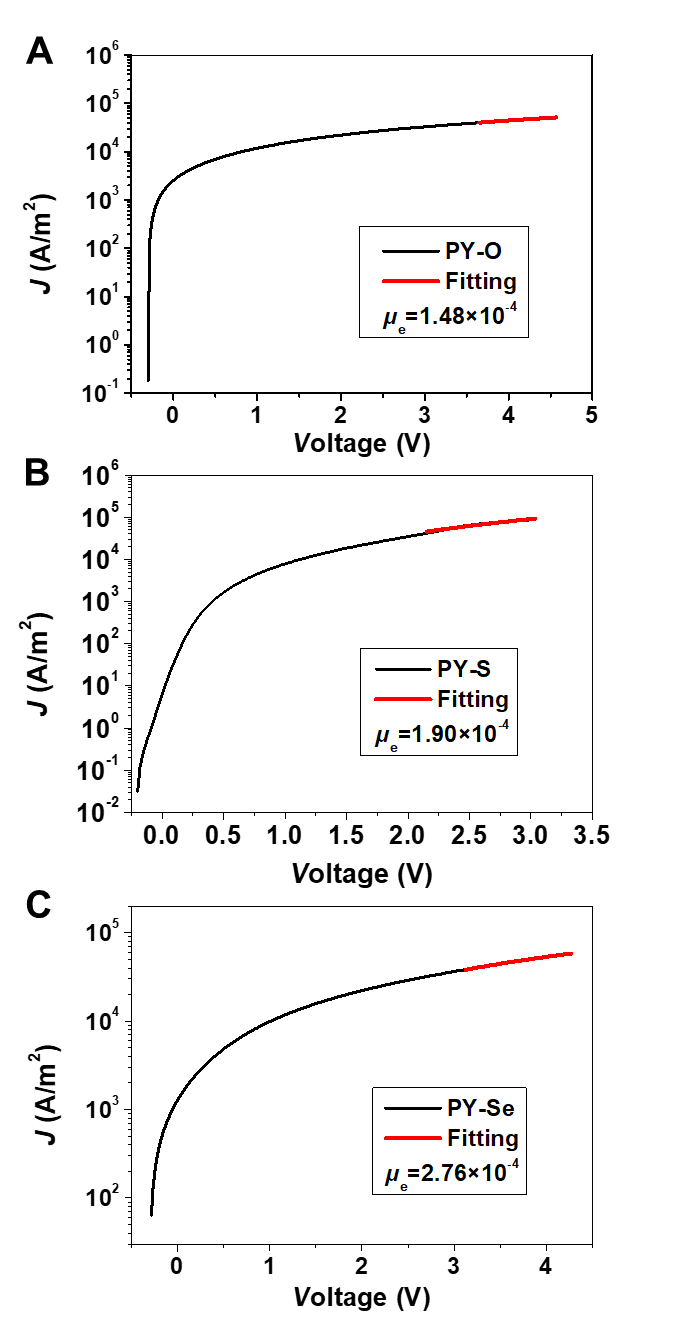


**Figure S9**. The dark *J-V* characteristics of electron-only mobility of (A) the pristine PY-O, (B) PY-S and (C) PY-Se films. The red lines represent the best fitting using the SCLC model. The inset mobility data are average values of four diodes.


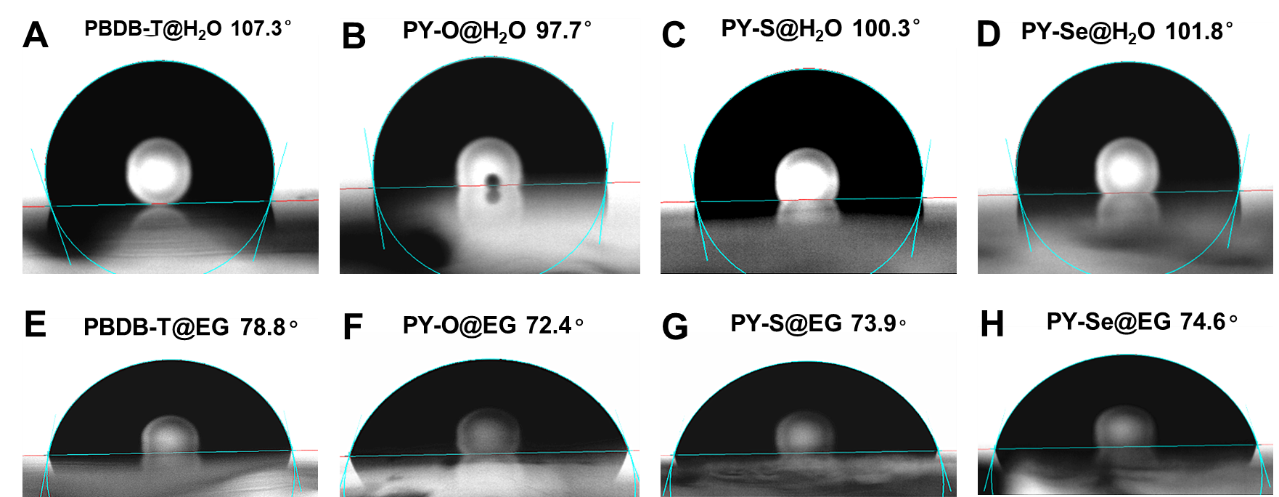


**Figure S10**. Photographs of water and EG droplets on the top surfaces of pristine PBDB-T and PYX (O, S, Se) films.

**Table S3**. Contact angles of water and ethylene glycol (EG) and their parameters for active layer materials.

| Organic layer | Contact angel  [H_2_O] (deg) | Contact angel  [EG] (deg) | Surface energy  (mN m^-1^) | χ  [PBDB-T, A] |
| --- | --- | --- | --- | --- |
| PBDB-T | 107.3 | 78.8 | 45.14 | n/a |
| PY-O | 97.7 | 72.4 | 33.37 | 0.88 |
| PY-S | 100.3 | 73.9 | 36.80 | 0.43 |
| PY-Se | 101.8 | 74.6 | 39.52 | 0.19 |

**Figure S11**. 2D GIWAXS patterns of (A) the neat PBDB-T film and (B) its scattering profiles of in-plane and out-of-plane.

**Table S4**. Summary of the extracted data from the GIWAXS patterns of the blends.

| Blend  (PBDB-T:Acceptor) | IP (100) | | OOP (010) | | |
| --- | --- | --- | --- | --- | --- |
|  | Q value  (Å^-1^) | Lattice spacing  (Å) | | FWHM  (Å^-1^) | Coherence length  (Å) |
| PY-O | 0.290 | 21.67 | | 0.370 | 16.98 |
| PY-S | 0.295 | 21.30 | | 0.361 | 17.40 |
| PY-Se | 0.295 | 21.30 | | 0.354 | 17.75 |

**Figure S12**. *J*-*V* curves of the OSCs based on PBDB-T:PY-O blends (3% CN) with different donor/acceptor weight ratios, under the illumination of AM 1.5 G at 100 mW cm^-2^.

**Table S5**. Photovoltaic parameters of the OSCs based on PBDB-T:PY-O (3% CN) blends with different donor/acceptor weight ratios, under the illumination of AM 1.5 G at 100 mW cm^-2^.

| D:A ratio | *V*_oc_ (V) | *J*_sc_ (mA cm^-2^) | FF (%) | PCE^[a]^ (%) |
| --- | --- | --- | --- | --- |
| 1:1 | 0.873 | 17.71 | 59.40 | 9.18(8.86) |
| 1:1.2 | 0.876 | 17.86 | 62.68 | 9.80(9.63) |
| 1:1.5 | 0.871 | 17.13 | 60.04 | 8.96(8.65) |

^a^The values in square bracket are the average PCE obtained from four devices.

**Figure S13**. *J*-*V* curves of the OSCs based on PBDB-T:PY-O blends (1:1.2, w/w) with different CN additive concentrations, under one sun illumination.

**Table S6**. Photovoltaic parameters of the OSCs based on PBDB-T:PY-O blends (1:1.2, w/w) with different CN additive concentrations, under one sun illumination.

| CN (vol%) | *V*_oc_ (V) | *J*_sc_ (mA cm^-2^) | FF (%) | PCE^[a]^ (%) |
| --- | --- | --- | --- | --- |
| 2.5 | 0.867 | 16.30 | 56.66 | 8.01(7.63) |
| 3.0 | 0.876 | 17.86 | 62.68 | 9.80(9.63) |
| 3.5 | 0.873 | 17.29 | 59.01 | 8.91(8.68) |

^a^The values in square bracket are the average PCE obtained from four devices

**Figure S14**. *J*-*V* curves of the OSCs based on PBDB-T:PY-Se blends (3% CN) with different donor/acceptor weight ratios, under the illumination of AM 1.5 G at 100 mW cm^-2^.

**Table S7**. Photovoltaic parameters of the OSCs based on PBDB-T:PY-Se (3% CN) blends with different donor/acceptor weight ratios, under the illumination of AM 1.5 G at 100 mW cm^-2^.

| D:A ratio | *V*_oc_ (V) | *J*_sc_ (mA cm^-2^) | FF (%) | PCE^[a]^ (%) |
| --- | --- | --- | --- | --- |
| 1:0.8 | 0.874 | 23.48 | 71.08 | 14.59(14.44) |
| 1:1 | 0.882 | 23.03 | 70.24 | 14.26(13.93) |
| 1:1.2 | 0.891 | 23.52 | 73.85 | 15.48 (15.16) |
| 1:1.5 | 0.885 | 23.26 | 69.91 | 14.47(14.29) |

^a^The values in square bracket are the average PCE obtained from four devices.

**Figure S15**. *J*-*V* curves of the OSCs based on PBDB-T:PY-Se blends (1:1.2, w/w) with different CN additive concentrations, under one sun illumination.

**Table S8**. Photovoltaic parameters of the OSCs based on PBDB-T:PYSe blends (1:1.2, w/w) with different CN additive concentrations, under one sun illumination.

| CN (vol%) | *V*_oc_ (V) | *J*_sc_ (mA cm^-2^) | FF (%) | PCE^[a]^ (%) |
| --- | --- | --- | --- | --- |
| 2.5 | 0.885 | 23.11 | 71.67 | 14.66(14.52) |
| 3.0 | 0.891 | 23.52 | 73.85 | 15.48 (15.16) |
| 3.5 | 0.889 | 23.46 | 71.96 | 15.00(14.90) |

^a^The values in square bracket are the average PCE obtained from four devices

**Figure S16**. Relevant absorption coefficient of the three all-polymer blends.

**Figure S17**. Normalized TPC data for the relevant devices. The illumination pulse intensity was 150 mW cm^−2^ (light pulse of 50 μs).


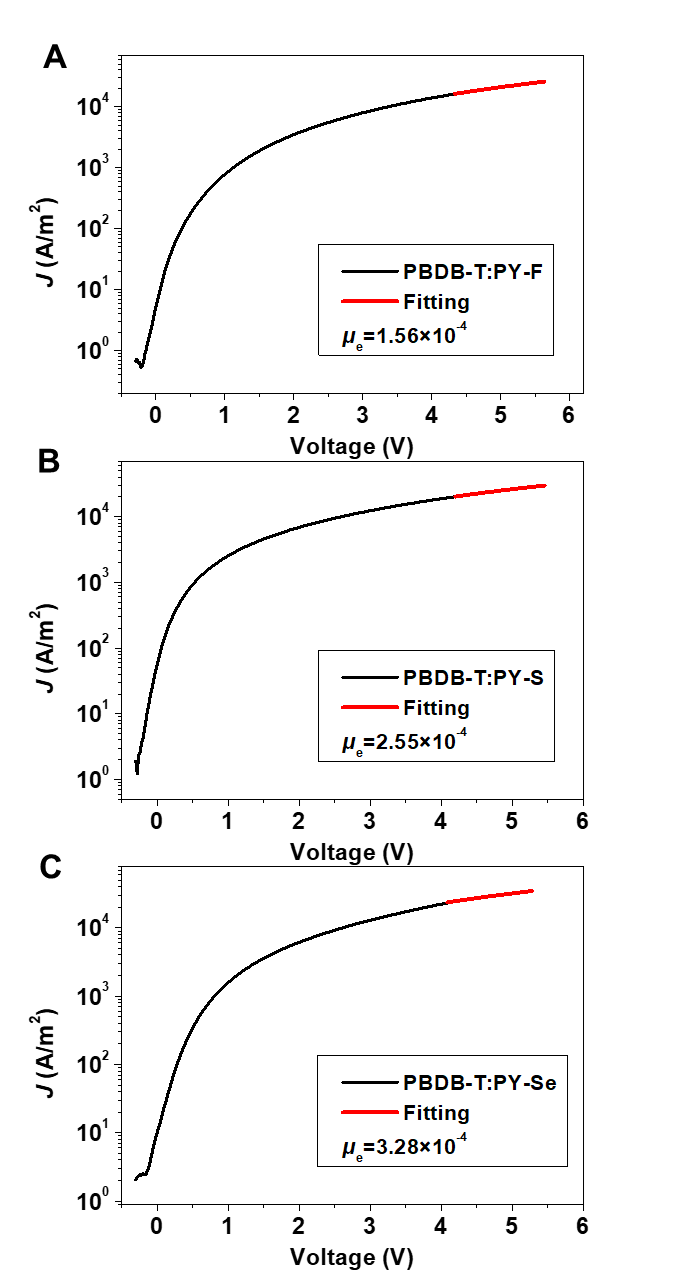


**Figure S18**. The dark *J-V* characteristics of electron-only mobility of (A) PBDB-T:PY-O, (B) PBDB-T:PY-S and (C) PBDB-T:PY-Se blend film. The red lines represent the best fitting using the SCLC model. The inset mobility data are average values of four diodes.


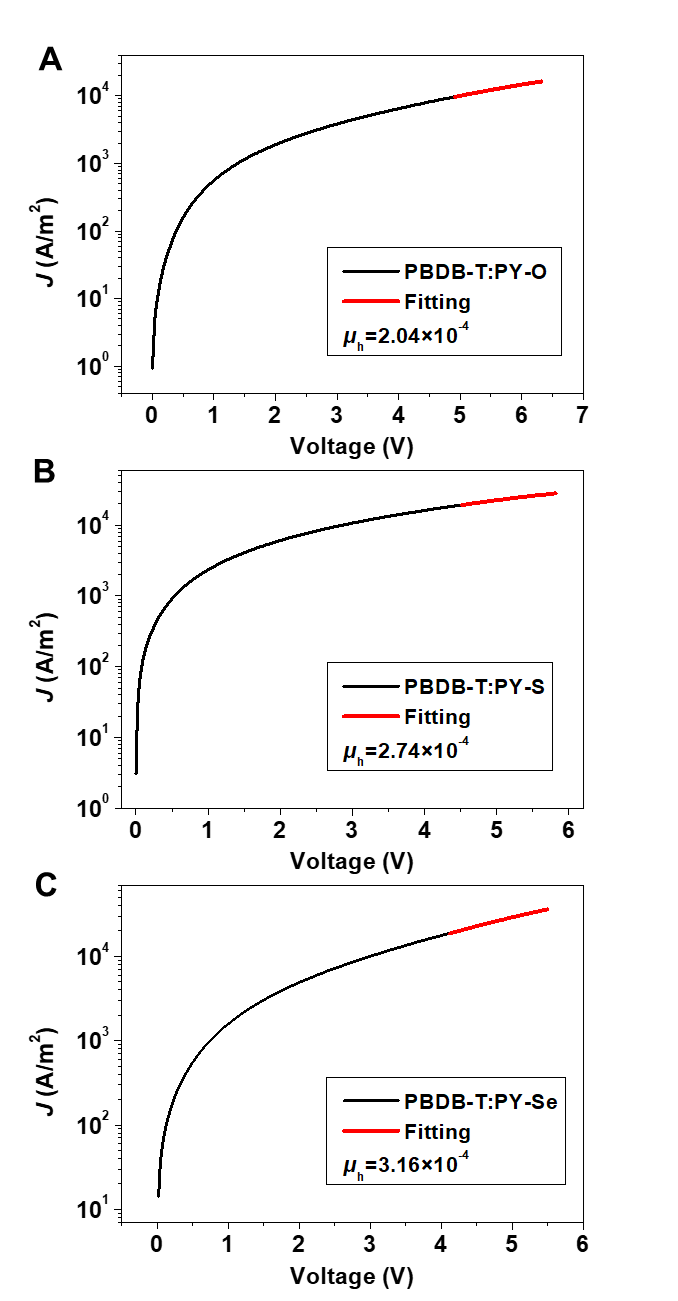


**Figure S19**. The dark *J-V* characteristics of hole-only mobility of (A) PBDB-T:PY-O, (B) PBDB-T:PY-S and (C) PBDB-T:PY-Se blend film. The red lines represent the best fitting using the SCLC model. The inset mobility data are average values of four diodes.


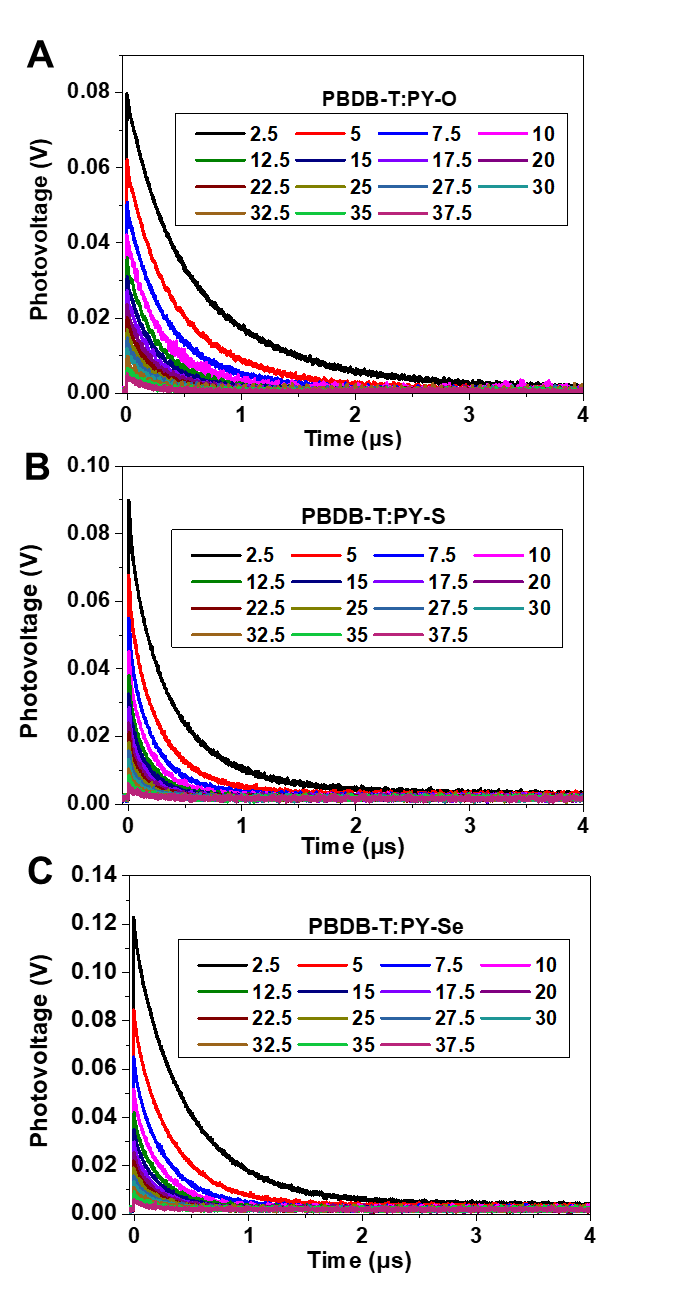


**Figure S20**. TPV measurements on the optimized (a) PBDB-T:PY-O, (b) PBDB-T:PY-S and (c) PBDB-T:PY-Se devices for light intensities of 0.15 to 2.50 sun. Transient photovoltage (TPV) measurements were used to analyze the recombination of free charges within the working devices by recording the transient voltage decay of the device under open circuit conditions under continuous illumination before a small perturbative light pulse was injected.


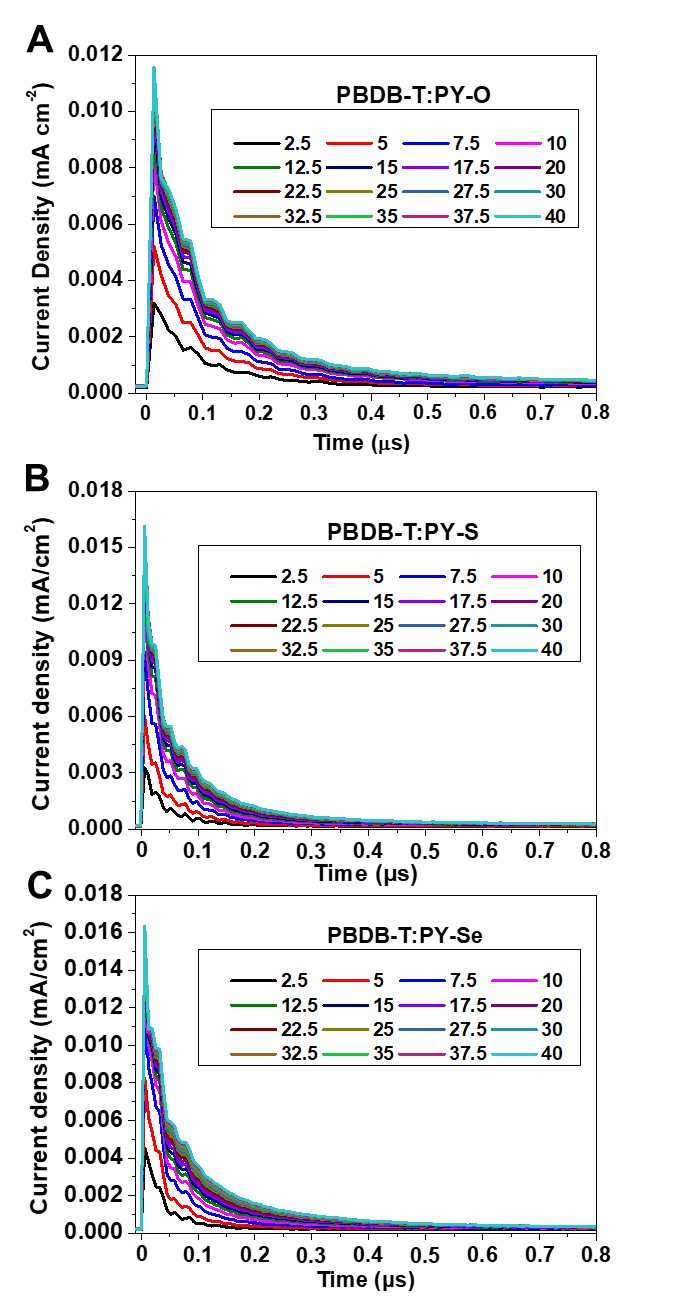


**Figure S21**. CE measurements on the optimized (a) PBDB-T:PY-O, (b) PBDB-T:PY-S and (c) PBDB-T:PY-Se devices for light intensities of 0.15 to 2.50 sun.

**Figure S22**. The external quantum efficiency of EL (EQE_EL_) values obtained from a home-built setup. Here the non-radiative voltage losses of these systems are 0.335 eV for the PBDB-T:PY-O, 0.327 eV for the PBDB-T:PY-S, and 0.323 eV for the PBDB-T:PY- Se, respectively.


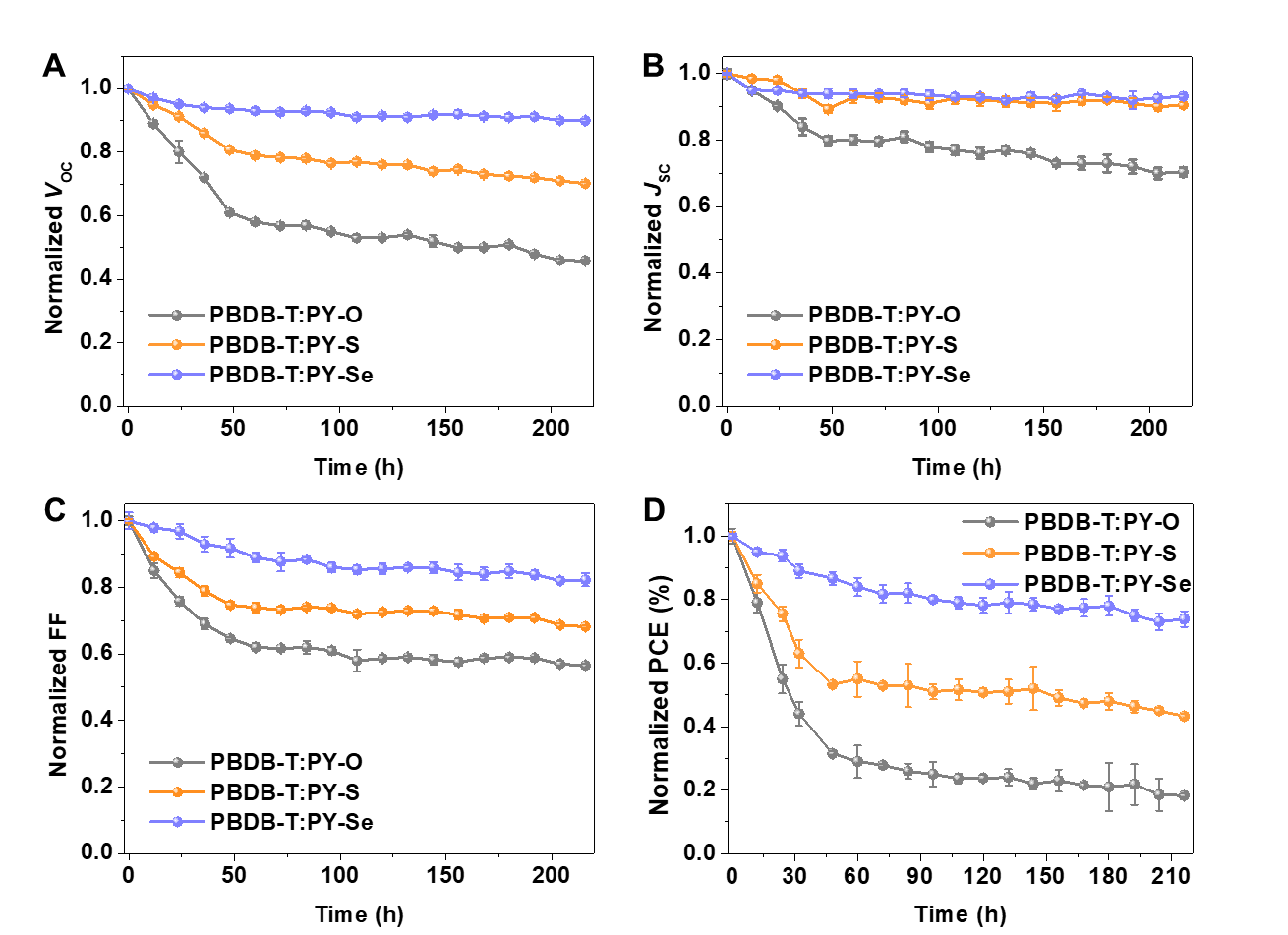


**Figure S23**. Changes of (A) normalized *V*_OC_, (B) normalized *J*_SC_, (C) FF and (D) normalized PCE losses over illumination time for PY-O, PY-S and PY-Se solar cells with each system for four cells.


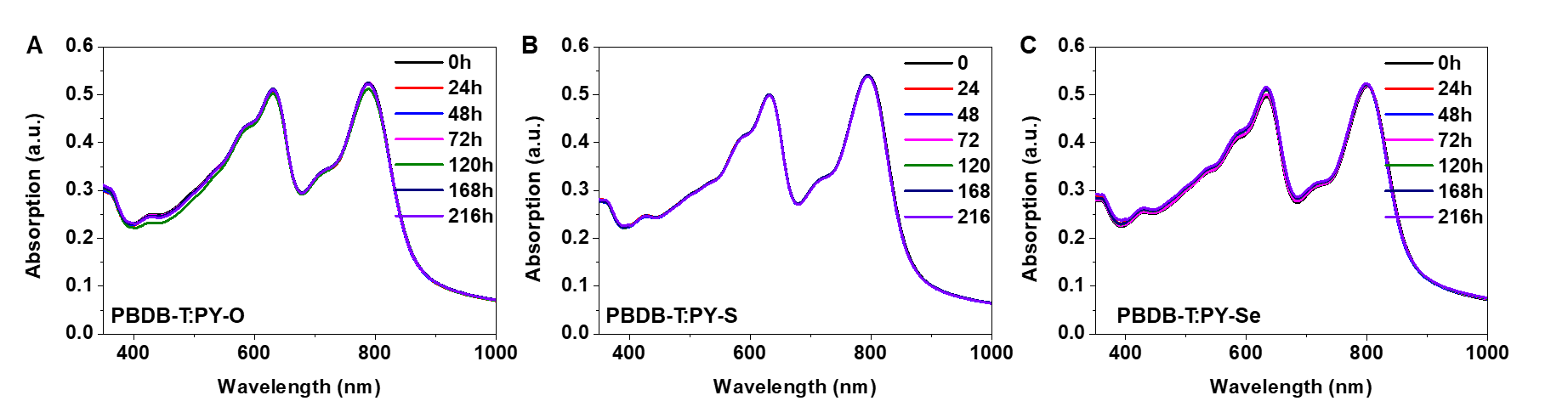


**Figure S24**. The UV-absorption spectra of PBDB-T:PY-O (A), PBDB-T:PY-S (B) and PBDB-T:PY-Se (C) blends as a function of illumination time at room temperature in the nitrogen glovebox.


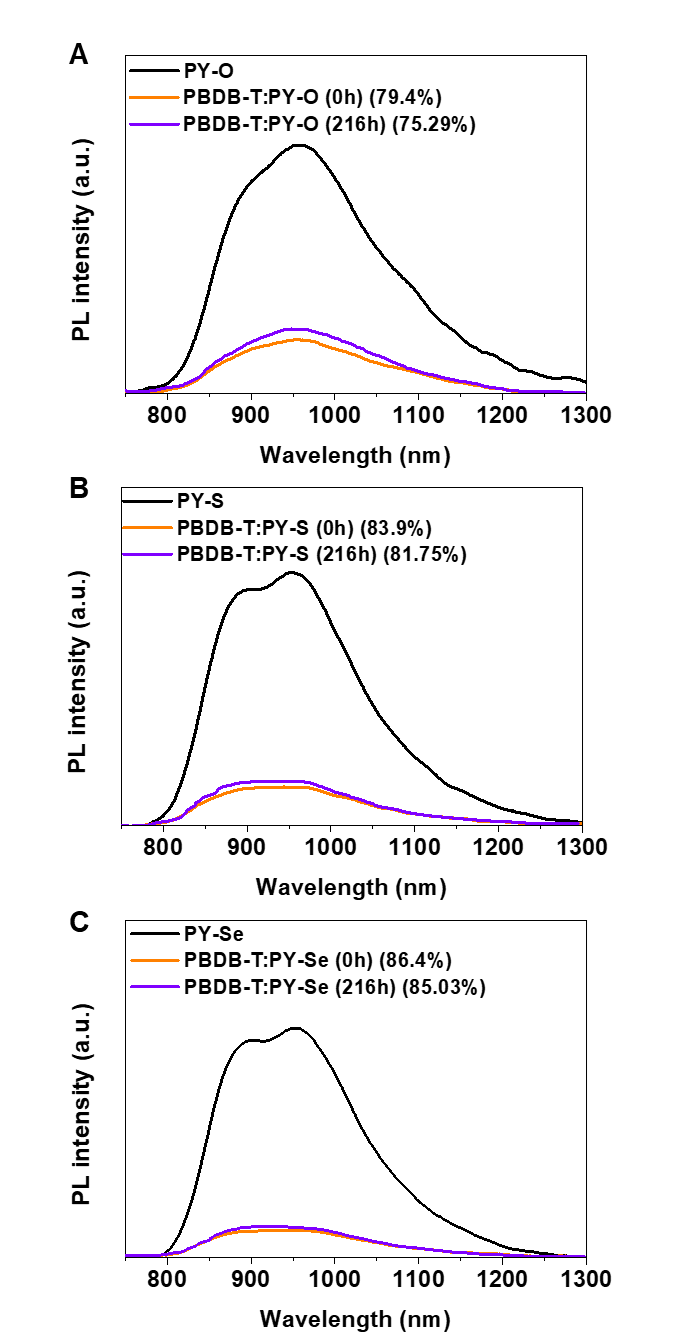


**Figure S25**. The PL spectra of PBDB-T:PY-O (A), PBDB-T:PY-S (B) and PBDB-T:PY-Se (C) blends as a function of illumination time at room temperature in the nitrogen glovebox. The changes in PL intensities under illumination for 216 hours are 75.29% for PBDB-T:PY-O, 81.75% for PBDB-T:PY-S, 85.03% for PBDB-T:PY-Se, respectively.


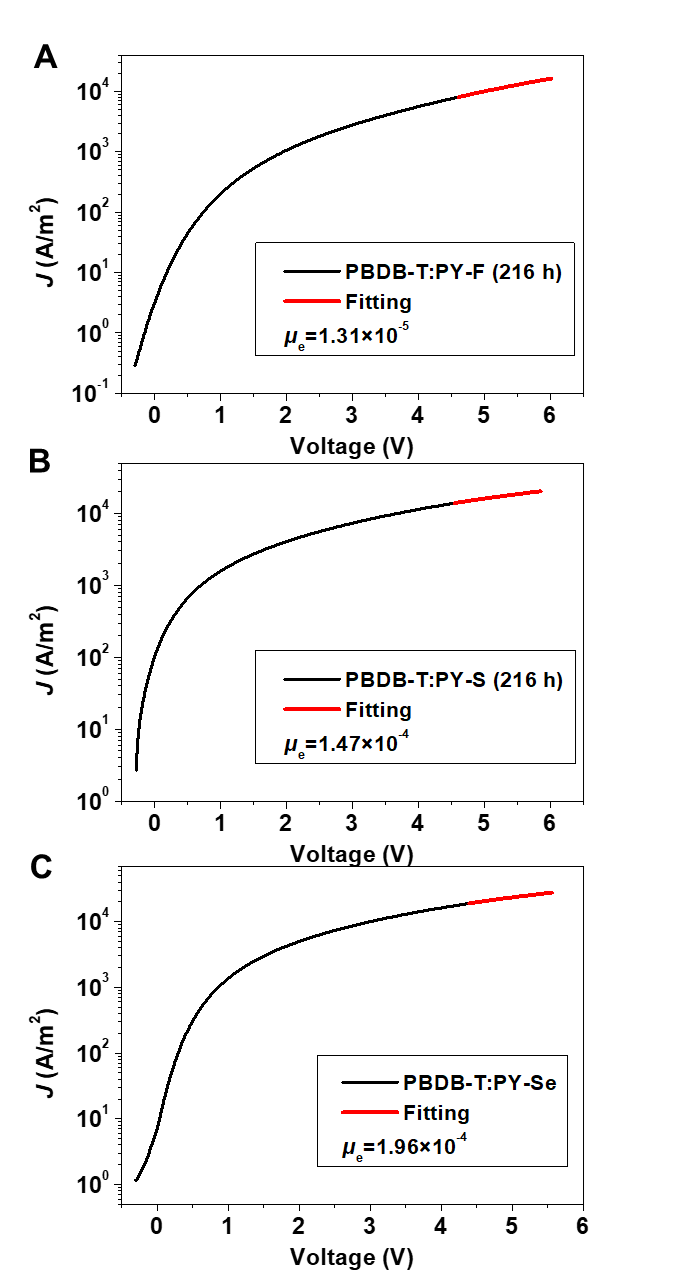


**Figure S26**. The dark *J-V* characteristics of electron-only mobility of (A) PBDB-T:PY-O, (B) PBDB-T:PY-S and (C) PBDB-T:PY-Se blend films under one sun illumination for 216 hours. The red lines represent the best fitting using the SCLC model. The inset mobility data are average values of four diodes.


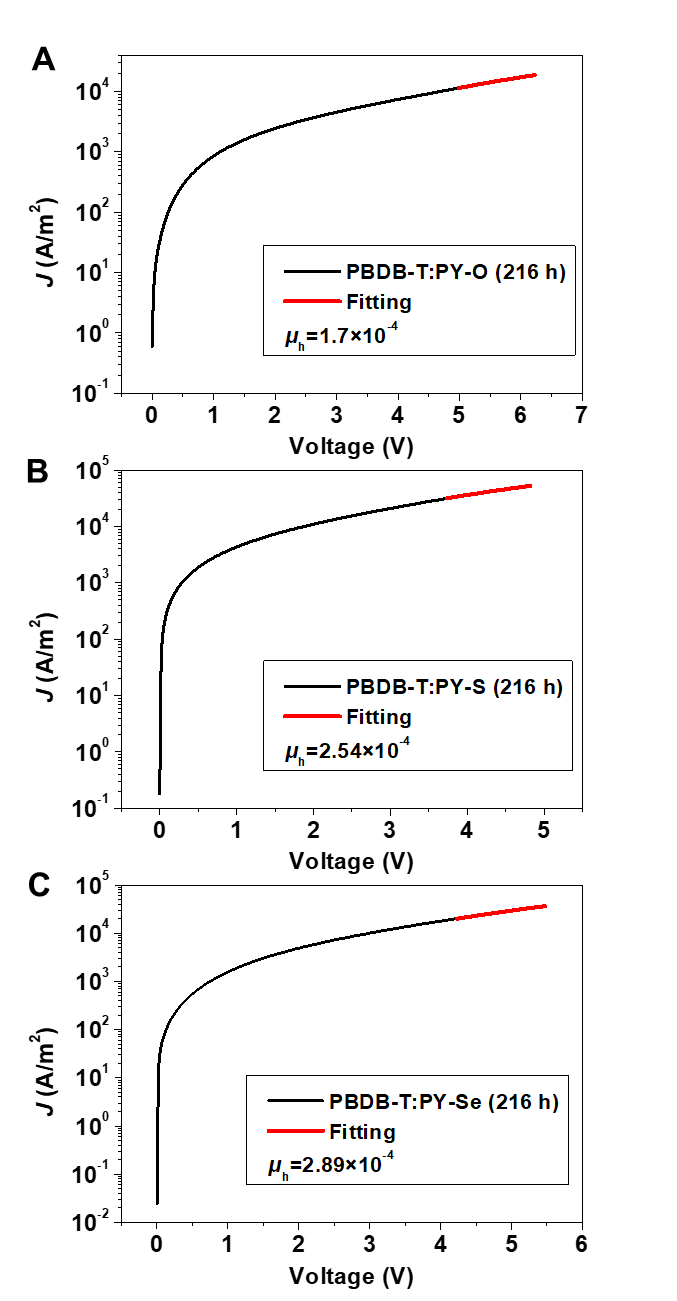


**Figure S27**. The dark *J-V* characteristics of hole-only mobility of (A) PBDB-T:PY-O, (B) PBDB-T:PY-S and (C) PBDB-T:PY-Se blend films under one sun illumination for 216 hours. The red lines represent the best fitting using the SCLC model. The inset mobility data are average values of four diodes.

**Table S9**. The properties of PBDB-T:PY-X (O, S, Se) devices under light soaking.

| Sample  (PBDB-T:P_A_s) | Time  (Light stability) | Quenching efficiency  (PL) | *µ*_e_  (×10^5^ cm^−1^) | *µ*_h_  (×10^5^ cm^−1^) | *µ*_e_/*µ*_h_ | R^a^ |
| --- | --- | --- | --- | --- | --- | --- |
| PY-F | 0 h | 79.4 | 1.56 | 2.04 | 0.76 | 2.29 |
|  | 216 h | 75.3 | 0.131 | 1.70 | 0.08 | 2.53 |
| PY-S | 0 h | 83.9 | 2.55 | 2.74 | 0.93 | 2.07 |
|  | 216 h | 81.8 | 1.47 | 2.54 | 0.58 | 2.18 |
| PY-Se | 0 h | 86.4 | 3.28 | 3.16 | 1.04 | 2.02 |
|  | 216 h | 85.0 | 1.96 | 2.89 | 0.68 | 2.08 |

^a^The carrier recombination order calculated from the TPV and CE measurements.


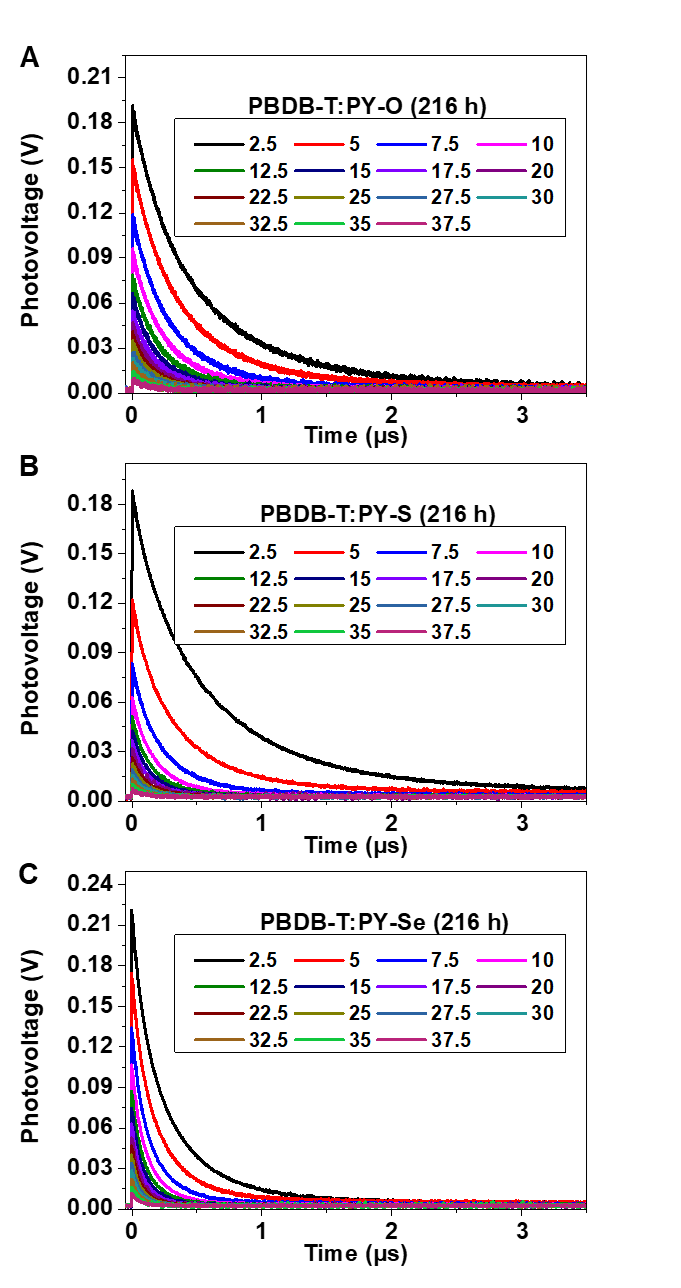


**Figure S28**. TPV measurements on the light-soaking devices of (a) PBDB-T:PY-O, (b) PBDB-T:PY-S and (c) PBDB-T:PY-Se for light intensities of 0.15 to 2.50 sun.


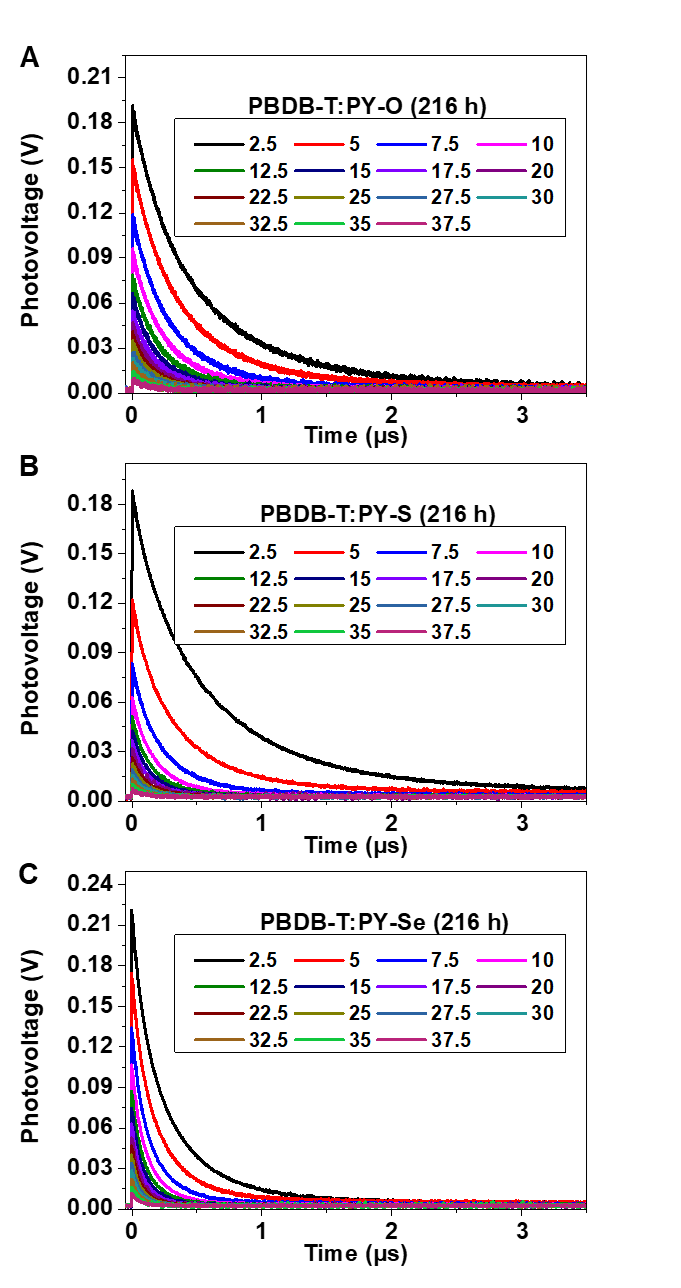


**Figure S29**. CE measurements on the light-soaking devices of (a) PBDB-T:PY-O, (b) PBDB-T:PY-S and (c) PBDB-T:PY-Se devices for light intensities of 0.15 to 2.50 sun.

**Figure 30**. Charge carrier lifetime τ, obtained from TPV, as a function of charge density n, calculated from CE under Voc conditions (from 0.15 to 2.50 suns). The devices were exposed after 216 hours under one sun illumination in N2-filled glovebox

**Table S10**. Mechanical properties of PBDB-T:PY-X (O, S, Se) films from strain-stress curves.

| Organic layer | Elastic modulus (MPa) | COS  % | Tensile strength (MPa) | Toughness  (J m^-3^) |
| --- | --- | --- | --- | --- |
| PBDB-T:Y5-C20 | 702.0±6.4 | 4.76±0.39 | 27.57±1.5 | 0.83±0.12 |
| PBDB-T: PY-O | 457.3±6.7 | 9.57±0.35 | 26.10±0.9 | 1.87±0.24 |
| PBDB-T: PY-S | 545.5±3.9 | 8.80±0.28 | 28.83±1.3 | 2.34±0.27 |
| PBDB-T: PY-Se | 542.9±4.2 | 8.70±0.42 | 33.84±1.6 | 2.22±0.19 |


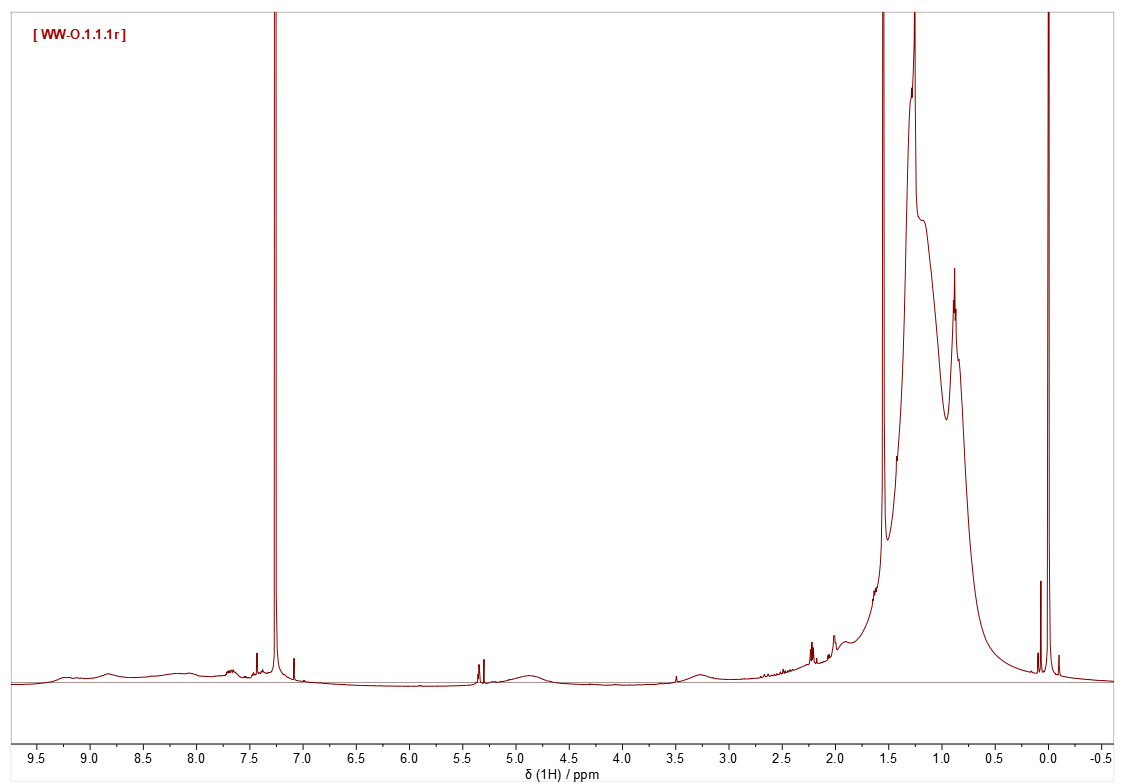


**Figure S31.** ^1^H-NMR spectrum of PY-O.


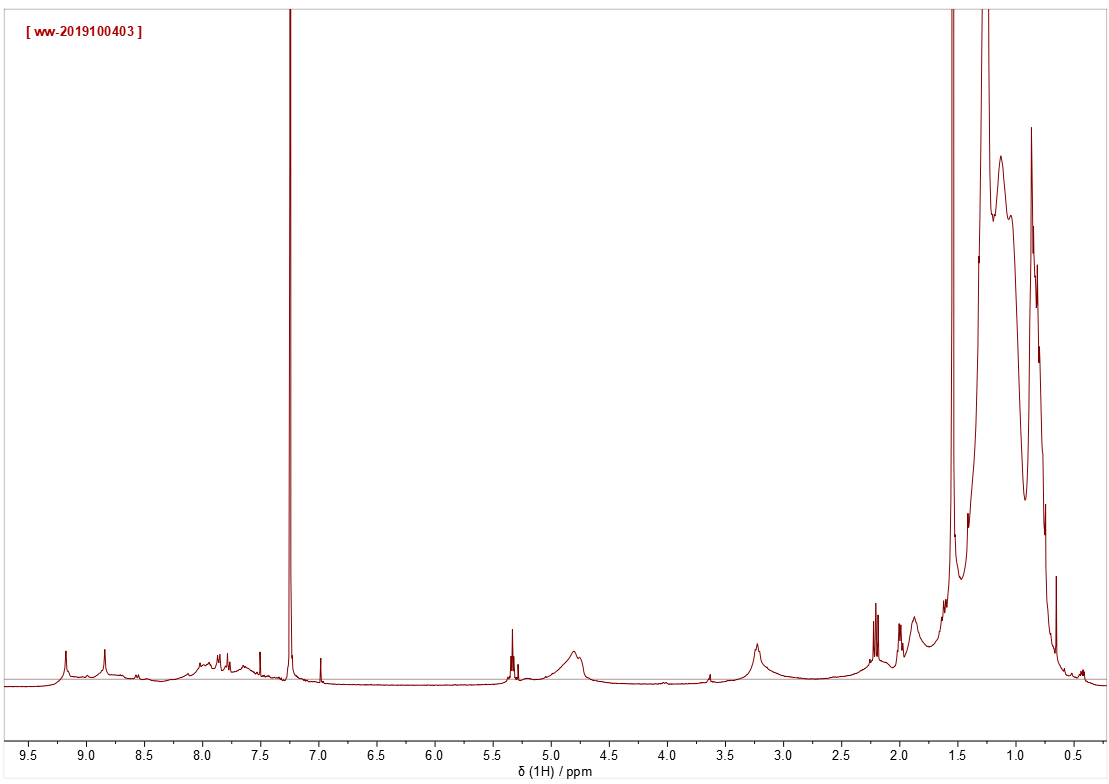


**Figure S32.** ^1^H-NMR spectrum of PY-S.


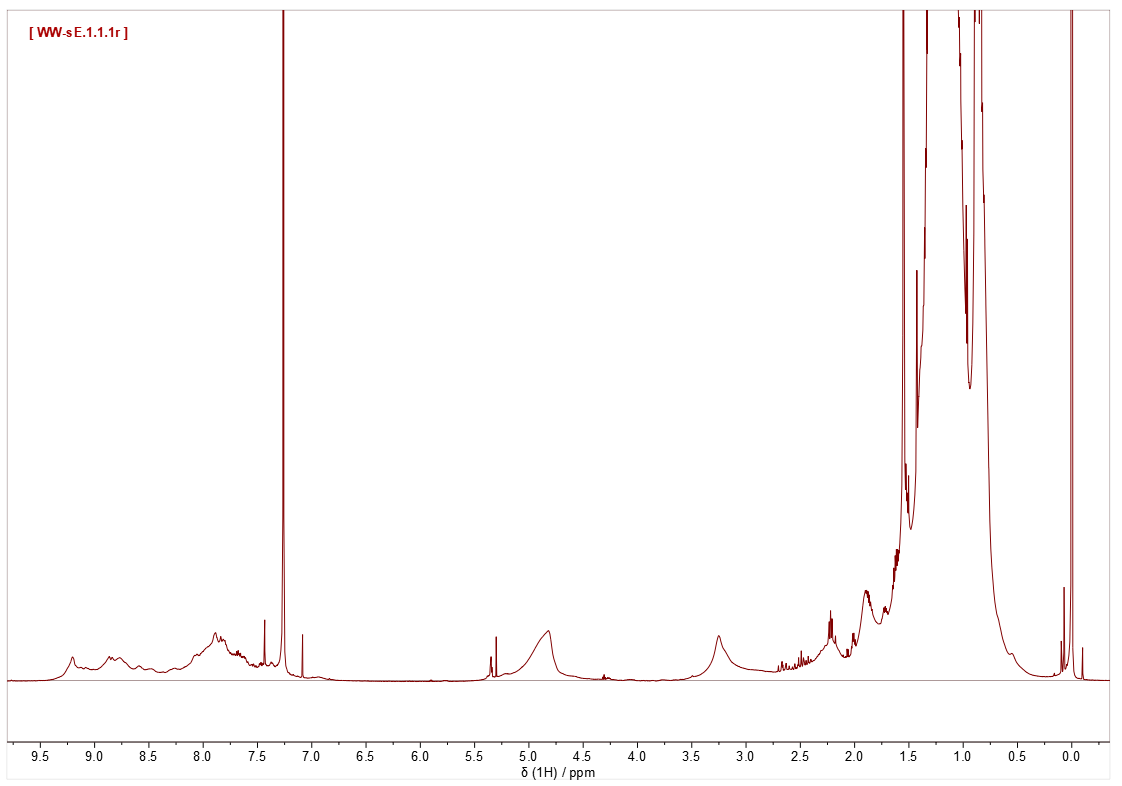


**Figure S33.** ^1^H-NMR spectrum of PY-Se.

**Reference**

1 Wu Q, Wang W and Wang T et al. High-performance all-polymer solar cells with only 0.47 eV energy loss. *Sci China Chem* 2020; ***63***: 1449.
